# Development of a Human iPSC Cardiomyocyte-Based Scoring System for Cardiac Hazard Identification in Early Drug Safety De-risking

**DOI:** 10.1016/j.stemcr.2018.11.007

**Published:** 2018-12-11

**Authors:** Ivan Kopljar, Hua Rong Lu, Karel Van Ammel, Martin Otava, Fetene Tekle, Ard Teisman, David J. Gallacher

**Affiliations:** 1 Global Safety Pharmacology, Non-Clinical Safety, Janssen Research & Development, A Division of Janssen Pharmaceutica NV, Turnhoutseweg 30, 2340 Beerse, Belgium; 2 Statistics and Decision Sciences, Quantitative Sciences, Janssen Research & Development, A Division of Janssen Pharmaceutica NV, Turnhoutseweg 30, 2340 Beerse, Belgium

**Keywords:** cardiomyocytes, stem cells, drug discovery and development, drug screening, cardiac safety, cardiac hazard, pharmacology, arrhythmia, torsade de pointes, hERG

## Abstract

Human induced pluripotent stem cell-derived cardiomyocytes (hiPSC-CMs) have emerged as a promising cardiac safety platform, demonstrated by numerous validation studies using drugs with known cardiac adverse effects in humans. However, the challenge remains to implement hiPSC-CMs into cardiac de-risking of new chemical entities (NCEs) during preclinical drug development. Here, we used the calcium transient screening assay in hiPSC-CMs to develop a hazard score system for cardiac electrical liabilities. Tolerance interval calculations and evaluation of different classes of cardio-active drugs enabled us to develop a weighted scoring matrix. This approach allowed the translation of various pharmacological effects in hiPSC-CMs into a single hazard label (no, low, high, or very high hazard). Evaluation of 587 internal NCEs and good translation to *ex vivo* and *in vivo* models for a subset of these NCEs highlight the value of the cardiac hazard scoring in facilitating the selection of compounds during early drug safety screening.

## Highlights

• Scoring system identifies different degrees of cardiac hazard
• Can be applied within R&D to cardiac safety screening of NCEs
• Controls and reference drugs are essential for development of scoring matrix
• Analysis can be applied to other *in vitro* drug safety assays

## Teaser

Kopljar and colleagues developed a scoring system to evaluate the potential cardiac hazard of drugs using a hiPSC-CM-based screening assay. This approach allows translation of various pharmacological responses in hiPSC-CMs into a single hazard label. Early evaluation of thousands of new chemical entities within drug discovery and development should enhance the development of new drugs with a favorable cardio-safety profile.

## Introduction

Early assessment of cardiac safety liabilities within drug discovery and development is essential to advance promising new chemical entities (NCEs) into clinical evaluation. As such, late-stage attrition due to cardiac safety could be mostly avoided, reducing the potential risk for participants in clinical studies and the costs of getting to this stage. The primary focus of cardiac safety within the current regulatory guidelines is to avoid drug-induced, potentially life-threatening arrhythmias such as torsades de pointes (TdP) (Gintant et al., 2016). TdP is associated with prolonged repolarization of the cardiac action potential, which is observed as prolongation of the QT interval of the electrocardiogram. Inhibition of the hERG channel (gene: *KCNH2*), a voltage-gated K^+^ channel that conveys the cardiac rapid delayed rectifier potassium current (I_Kr_), is the main mechanism associated with drug-induced QT prolongation.

Cardiac action potentials are mediated by multiple ionic currents that can alter the cardiac excitability and function of the heart. In addition to inhibiting hERG, drugs can influence electrophysiological function via various cardiac targets such as cardiac sodium current (I_Na_), calcium current (I_Ca_), pacemaker current (I_f_), ATP-sensitive potassium currents (I_KATP_), slow delayed potassium current (I_Ks_), and calcium-handling proteins, such as Na^+^/K^+^ ATPases and ryanodine receptors. Beyond QT prolongation, these additional pharmacological actions can result in drug-induced cardiac liabilities such as QT shortening and QRS widening, which are also associated with bradycardia and cardiac arrest, and non-TdP ventricular tachycardia or ventricular fibrillation. These cardiac liabilities not related to prolongation of repolarization also need to be considered during cardiac safety evaluation in pharmaceutical research and development (R&D) (Lu et al., 2008, Lu et al., 2010).

During drug discovery, early potential hazard identification is generally evaluated through binding or functional assays for hERG and other ion channels. However, these assays lack the complexity of an integrated cardiac cellular system (e.g., cardiomyocytes) and provide only indirect insights into potential cardiac electrophysiological actions of compounds. Alternative and/or follow-up studies that do possess the required complex interplay of different ion channels are generally based on animal models. These models have a low throughput, are labor and cost inefficient, and raise concerns about species translation and consistency with the 3R's (reduce, replace, and refine) concept of animal use. Recently, human induced pluripotent stem cell-derived cardiomyocytes (hiPSC-CMs) have emerged as a promising human-derived cardiac *in vitro* platform that can be used in preclinical safety evaluation. The potential of hiPSC-CMs has been recognized and supported by the Comprehensive *In Vitro* Proarrhythmia Assay (CiPA) initiative, with the aim to reshape the existing regulatory guidelines to help identify the torsadogenic (proarrhythmic) risk of drugs (Colatsky et al., 2016). Consequently, hiPSC-CMs have been extensively evaluated mainly for drug-induced QT prolongation and TdP risk. Nevertheless, hiPSC-CMs represent a relevant cardiac model containing the ionic currents that shape the cardiac action potential. This could allow cardiac hazard identification for different pharmacological mechanisms, beyond hERG or QT prolongation, and positioning of hiPSC-CMs as a versatile early preclinical safety de-risking screening tool. One of the routinely applied measurement methods is calcium transient imaging using calcium-sensitive indicators (Broyles et al., 2018). Intracellular calcium transients reflect the rise and decay of cytosolic Ca^2+^ during a cardiac action potential. Imaging of the internal calcium transients has been shown to reflect action potential duration (APD) and arrhythmic events in hiPSC-CMs (Spencer et al., 2014). Early afterdepolarization (EAD)-like events in hiPSC-CMs, detected with calcium imaging in the form of additional (abnormal) calcium spikes or bursts during a single calcium transient cycle, may represent a potential surrogate for TdP risk in humans. Studies have shown the translational value of the calcium transient-based hiPSC-CM (CTCM) assay by evaluating drugs with known cardiac liabilities in humans (Bedut et al., 2016, Dempsey et al., 2016, Lu et al., 2015, Rast et al., 2015, Watanabe et al., 2017, Zeng et al., 2016). Furthermore, hiPSC-CMs are suggested to show more relevant pharmacological responses in comparison with existent hERG and certain non-human APD or isolated heart assays (Takasuna et al., 2017).

The CTCM assay allows for medium- to high-throughput evaluation of hundreds to thousands of NCEs per year. However, one of the main challenges that remains is assessment of the translational potential of hiPSC-CM assays and implementation for hazard identification and decision-making during (early) preclinical drug discovery. Therefore, a straightforward approach is needed for interpretation of large datasets from hiPSC-CMs. Ideally, the high-throughput CTCM data are converted into an integrated score that can then be used to rank compounds based on hazard level as a function of concentration. The present study illustrates the scientific approaches used to develop and validate a hazard scoring system and conveys opportunities to apply such an approach in other laboratories, and to other technologies and models in early R&D safety assessment.

## Results

### Fundamentals of Cardiac Hazard Scoring

**Figure 1:**
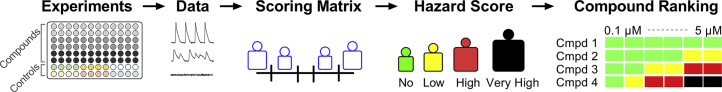
Concept of Cardiac Hazard Identification Compounds were tested in hiPSC-CMs using a calcium fluorescence imaging assay. Effects on hiPSC-CMs are evaluated through different parameters, which are then fed into a scoring matrix that translates the data into a hazard level. This approach allows a concentration-dependent hazard identification and ranking of drug candidates.

The goal of our current work was to translate pharmacological effects on hiPSC-CMs using a phenotypic readout (e.g., calcium imaging) into a cardiac hazard score for NCEs being considered for further drug development (Figure 1). Cardiac hazard scoring for the tested compound at a given concentration was differentiated into the following classes (color labels): “no” (green), “low” (yellow), “high” (red), and “very high” (black). No hazard labeling indicates compound effects within the vehicle variability. Low hazard suggests effects outside of the vehicle variability, with likely no or limited risk. High hazard suggests a strong concern that could potentially lead to cardiac adverse effects in the clinic, whereas the very high hazard identifies cardiac arrhythmias, such as torsadogenic risk (e.g., TdP), which could potentially lead to life-threatening events. The respective hazard class was determined by a cumulative scoring of individual parameters using a scoring matrix, which was based on the following three fundamental components: (1) selection of the relevant parameters, (2) defining the cutoffs for level of effect, and (3) defining weighted points per parameter for level of hazard potential.

### Characteristics of Calcium Transients in hiPSC-CMs

**Figure 2:**
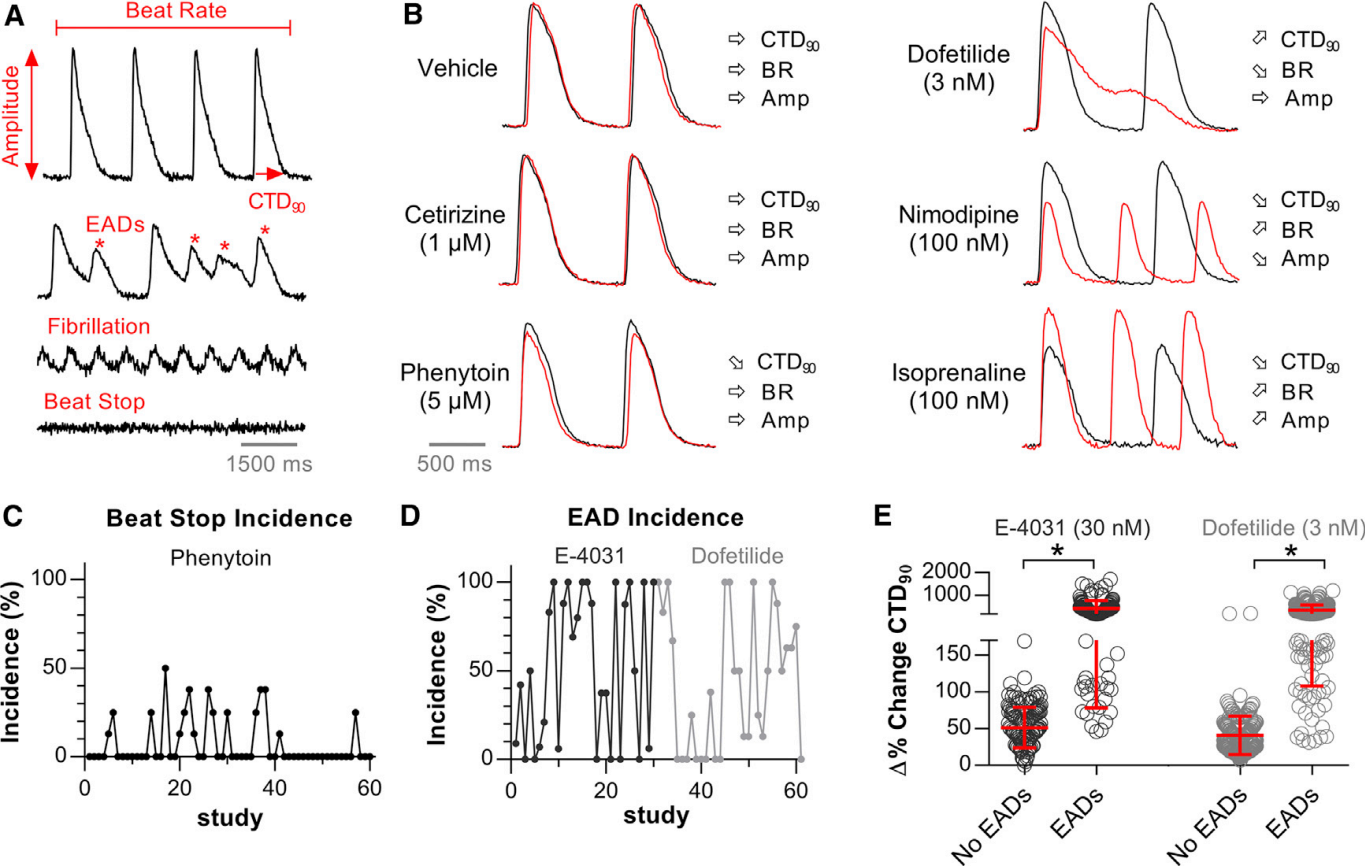
Characteristics and Pharmacological Response of hiPSC-CMs (A) Representative calcium transient recording allowing evaluation of CTD_90_, BR, and Amp. Abnormal functioning of hiPSC-CMs was observed in the form of EAD-like events, fibrillation-like events, and beating arrest. (B) Representative calcium transients showing the 30-min effect (red tracing) of vehicle and control drugs compared with their respective baseline (black tracing). E-4031 effects (not shown) were similar to dofetilide. (C) Incidence of beat stop (% of wells) after a 30-min incubation with phenytoin at 5 μM. Each study represents n = 4–8 independent experiments. (D) Incidence of EADs (% of wells) developed during a 30-min incubation with E-4031 (30 nM) or dofetilide (3 nM). (E) Relationship between the size of CTD_90_ prolongation and EAD occurrence for E-4031 (EAD, n = 150; no EAD, n = 113) and dofetilide (EAD, n = 179; no EAD, n = 193). n represents independent experiments. Error bars represent SD. ^∗^p < 0.0001, Mann-Whitney test.

The CTCM assay is a medium- to high-throughput screening tool used for early cardiac safety testing of compounds. Fluorescence imaging of intracellular calcium dynamics (i.e., calcium transients) enabled the evaluation of the spontaneous beating properties of hiPSC-CMs (Figure 2A) using the following parameters: (1) calcium transient duration at 90% of decay following the peak amplitude (CTD_90_), (2) beat rate (BR) representing the number of calcium transients (i.e., beats) per minute, and (3) amplitude (Amp), representing the difference between the minimum and the maximum (peak) calcium signal. Figure S1 shows the baseline CTD_90_ and BR values for 23,183 individual experiments used for this study. Although the histograms reflect a Gaussian distribution, the range of CTD_90_ and BR values also showed the expected heterogeneity in hiPSC-CMs, reflecting a mixture of different cell types (e.g., ventricular, atrial, and nodal-like) within the syncytium and batch-to-batch and experimental variability. Furthermore, we used uncorrected CTD_90_ values since BR-dependent correction of CTD_90_ was not optimal for our purposes (Figure S2).

For quality control of hiPSC-CMs, each test plate (i.e., 96 unique experiments) contained vehicle treatments together with several control drugs (cetirizine, phenytoin, isoprenaline, nimodipine, and dofetilide or E-4031). These drugs represent different pharmacological classes and were used as a pharmacological reference set for the development of the hazard scoring system. Cetirizine is a true negative control without any clinical cardiac liabilities. Phenytoin is a sodium channel blocker for antiepileptic treatment, with class Ib antiarrhythmic properties, but is not known to be overtly proarrhythmic in the clinic. Nimodipine is a calcium channel antagonist and isoprenaline is a beta-adrenergic agonist. Dofetilide, a hERG blocker associated with QT prolongation and proarrhythmic TdP risk in humans, was also evaluated. E-4031, an experimental hERG blocker, was used as a second agent in this class. Effects on hiPSC-CMs were evaluated after a 30-min incubation period and normalized against the respective baseline recording, yielding a Δ% change in CTD_90_, BR, and Amp.

### Scoring Matrix: Relevant Parameters for Cardiac Hazard Identification

Selection of parameters is one of the crucial aspects of the scoring matrix. The parameters need to reflect relevant changes related to pharmacological effects and preferentially differentiate the severity and direction of effect. CTD_90_, BR, and Amp (Figure 2A) were selected as primary parameters to quantify changes in calcium transients. CTD_90_ is the main parameter, which reflects both APD prolongation and shortening by compounds, and is used as a surrogate for QT interval changes. BR is secondary with respect to effects on CTD_90_. However, it is more relevant for pharmacological classes such as adrenergic agonists, which increase the BR of cardiomyocytes. On the other hand, strongly decreased BR can be related to bradycardic actions or effects on action potential propagation. Amp is a parameter that can give insights regarding effects on internal calcium handling. For example, adrenergic stimulation (e.g., isoprenaline) or Ca^2+^ channel agonists (e.g., BAYK8644) will increase the Amp, whereas Ca^2+^ channel antagonists (e.g., nimodipine) will strongly decrease the Amp. In general, various pharmacological mechanisms evoke a specific (directional) effect on the three parameters (Figure 2B).

In addition to the primary beating parameters, we introduced additional parameters into the scoring matrix in the form of “beat stop,” “fibrillation-like” events, and “EADs” (Figure 2A). Beat stop is a multi-purpose parameter since it can reflect various effects on hiPSC-CMs. For example, compounds (at high concentrations) can have strong effects on cardiac electrophysiology that can lead to a beating arrest of the syncytium. Although there would be no information on primary parameter effects, beating arrest indicates a relevant pharmacological action in hiPSC-CMs. However, in certain cases an incidence of beat stop (≤50%) can be observed with relatively cardiac-safe compounds such as phenytoin (Figure 2C), due to weak effects on the sodium current, which can alter the spontaneous beating propagation of hiPSC-CMs. Therefore, we introduced three zones for beat stop incidence (based on percentage of wells with beat stop) to differentiate the level of potential hazard of compounds (Figure 4A). A fibrillation-like phenotype can occur when normal propagation of beating becomes discontinuous, leading to impulse reentry and a fibrillation-like state, as reflected by small, rapid-rate calcium transients. Finally, hiPSC-CMs can develop arrhythmic beating, where additional calcium spikes are observed during a calcium transient or before the following one has initiated (i.e., EAD-like events). This behavior is observed with QT-prolonging drugs, which display proarrhythmic risk and can cause life-threatening TdP in humans. Indeed, E-4031 and dofetilide, positive controls for I_Kr_ (hERG) inhibition, provoked such EAD-like events in hiPSC-CMs, but the incidence varied between plates and studies (Figure 2D). Furthermore, we observed a clear overlap in Δ% CTD_90_ effect ranges between the experimental populations without EADs and those with EADs (Figure 2E). This implies that a cutoff for strong CTD_90_ prolongation can be used to distinguish potential QT prolongation that is not necessarily associated with TdP risk. Therefore, we annotated EADs as a separate parameter instead of predicting EADs using changes in CTD_90_. Hence, studies showing EAD observations are uniquely categorized as very high (black) hazard.

### Scoring Matrix: Defining Cutoffs and Weighted Points

**Figure 3:**
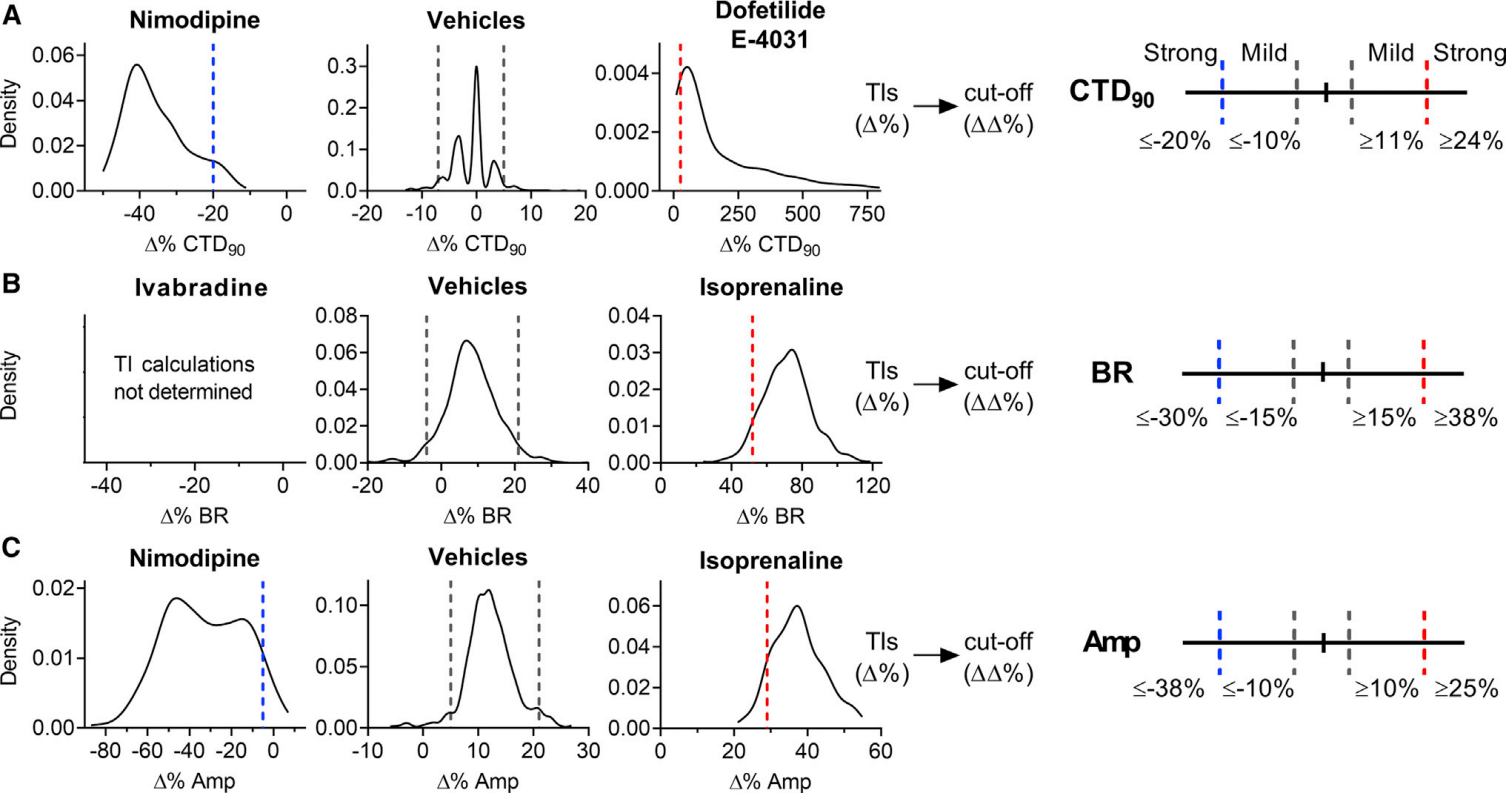
Determination of Cutoffs Using TIs to Develop the Scoring Matrix Density plots show the TIs (dashed lines within graphs) for vehicles and positive controls to determine bidirectional cutoffs for (A) CTD_90_, (B) BR, and (C) Amp. TIs on vehicles were applied to aid the process of defining the no effect cutoffs. TIs (Δ%) from control drugs were corrected for vehicle offset to determine the cutoffs (ΔΔ%). Vehicle (n = 639), isoprenaline (0.1 μM; n = 357), nimodipine (0.1 μM; n = 323), E-4031 (30 nM), and dofetilide (3 nM) were combined (n = 387), ivabradine (0.1 μM; n = 6). n indicates the number of independent experiments.

The next part of the scoring matrix was the determination of cutoffs between the different effect zones per parameter (Figure 3). The “no effect” zone represents changes in a parameter that are likely within vehicle variability, whereas “mild” and “strong” zones are differentiated bidirectionally (e.g., CTD_90_ shortening and prolongation). The cutoff values are net changes (ΔΔ% changes versus baseline and vehicle) that are based on the statistical tolerance intervals (TIs) (Δ% changes versus baseline). TIs indicate an interval where, with a certain confidence level, a specified proportion of a sampled population falls. Vehicle treatments showed low variability in Δ% change for CTD_90_, BR, and Amp with respect to baseline (Figure 3). Such low variability should facilitate the defining of the cutoffs and the development of a robust scoring system. The width of the TIs of vehicles was used to characterize the vehicle effects for each plate and assisted the scientific framework defining the two-sided cutoffs to determine the no effect zone for all parameters. Strong bidirectional cutoffs (Figure 3) were based on the calculation of TIs in numerous studies with control drugs (E-4031, dofetilide, nimodipine, and isoprenaline) and determined by correcting (per parameter) the respective TI with centralized TI windows of vehicles. Cutoffs defining mild changes in CTD_90_ were determined through an iterative optimization process (Figure S3). Evaluation of negative controls, QT-prolonging and QT-shortening drugs, allowed us to determine the TIs for effects on CTD_90_ while minimizing false positive scoring. The cutoff for strong BR decrease was based on the ΔΔ% change in BR by ivabradine (a reference drug) at 0.1 μM, a concentration at which the I_f_ current is inhibited without additional effects on hERG (half maximal inhibitory concentration [IC_50_] of approximately 2.4 μM).

**Figure 4:**
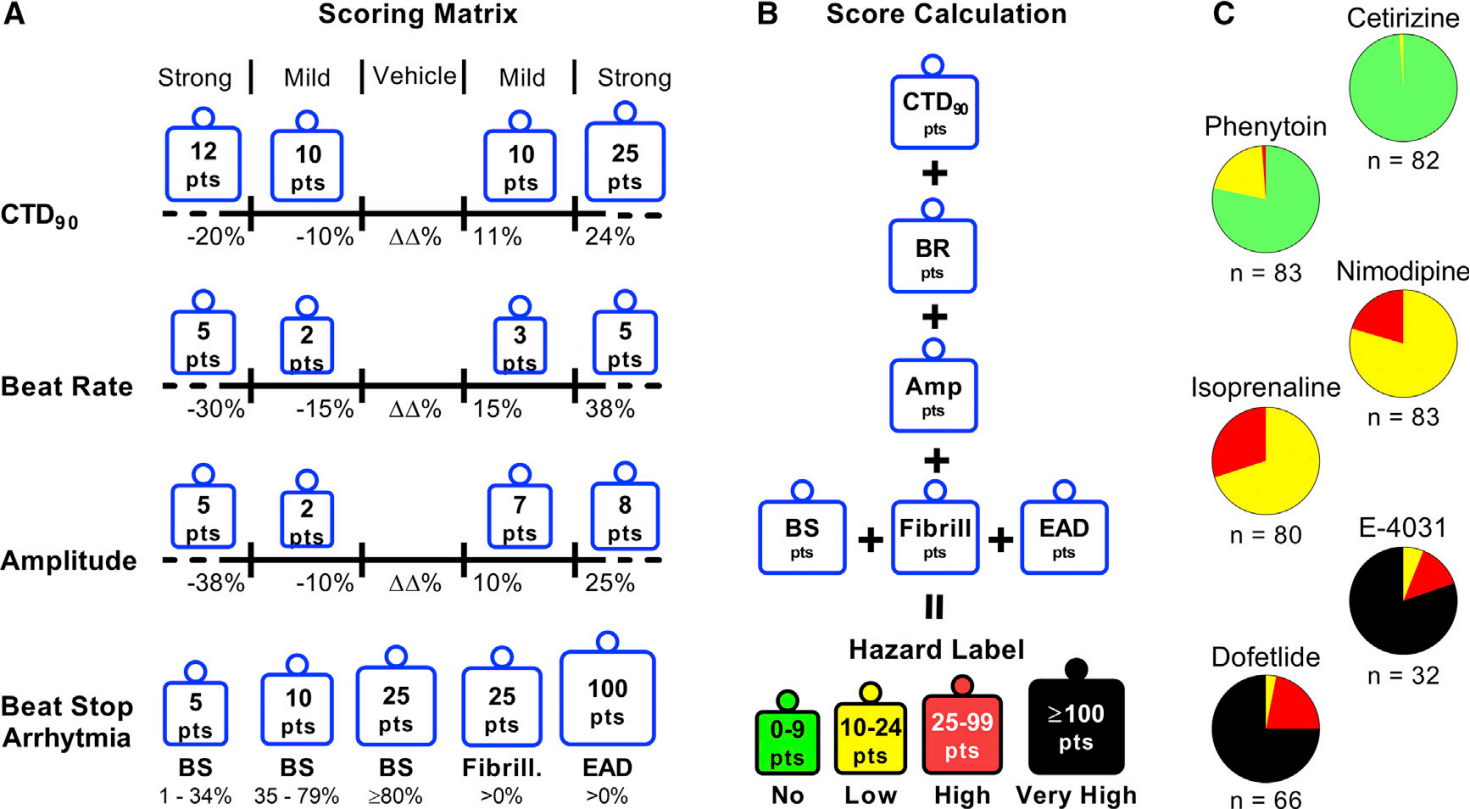
Development of the Scoring Matrix and Calculation Algorithm for Hazard Identification (A) The scoring matrix represents a points card where for each parameter a weighted score is given dependent on the size and direction of the ΔΔ% effect. (B) Calculation of hazard level is done through a sum of points across all parameters. (C) Scoring of controls over multiple studies: cetirizine (1 μM), phenytoin (5 μM), isoprenaline (0.1 μM), nimodipine (0.1 μM), E-4031 (30 nM), dofetilide (3 nM). n indicates the number of studies, each study contained 4–8 independent experiments.

The last part of the scoring matrix was the inclusion of a weighted points algorithm to differentiate various levels of cardiac hazard (Figure 4). Defining the weighted points was based on the relevance of a parameter (and direction of effect), together with an expected hazard identification for various pharmacological mechanisms of action. The sum of all weighted parameters resulted in a total hazard score that could be translated into a specific hazard label (Figure 4B). Here, an iterative approach (Supplemental Experimental Procedures and Figure S4) was applied to optimize the weighted points and the overall score ranges associated with the different hazard labels.

### Validation of the Hazard Scoring System Using Controls and Reference Drugs

Next, the optimized hazard scoring (labeling) was validated on all studies done with positive controls and reference drugs. It is important to note that the control drugs (an initial smaller set used in Figure 3) and the reference drugs (subset) were used for the development and optimization process of different parts of the scoring system. On the other hand, the validation part represents the overall outcome of the scoring system on all studies done within this analysis. However, it is recommended to use a validation set that is different from the reference (training) set of drugs. Figure 4C shows the hazard distribution for control drugs tested over numerous studies. Cetirizine at 1 μM (16-fold free plasma concentration [free C_max_]) as a negative control was almost exclusively identified as no hazard (99%), indicating that false positive hazard labeling is very rare. Figure S5 illustrates the influence of varying cutoffs for CTD_90_ on hazard scoring of cetirizine, indicating the importance of cutoff determination using a combination of statistical analysis and experimental evaluation. The sodium blocker phenytoin at 5 μM (free C_max_) was mainly identified as no hazard (78%) or low hazard (20%), as expected. Nimodipine at 0.1 μM (17-fold free C_max_) and isoprenaline at 0.1 μM are both cardio-active drugs that were identified within the low (80% and 70%, respectively) and high hazard zones. Dofetilide at 3 nM (2-fold free C_max_) was correctly identified as very high (80%) or high (14%) hazard. Similar results were observed with E-4031 at 30 nM.

**Figure 5:**
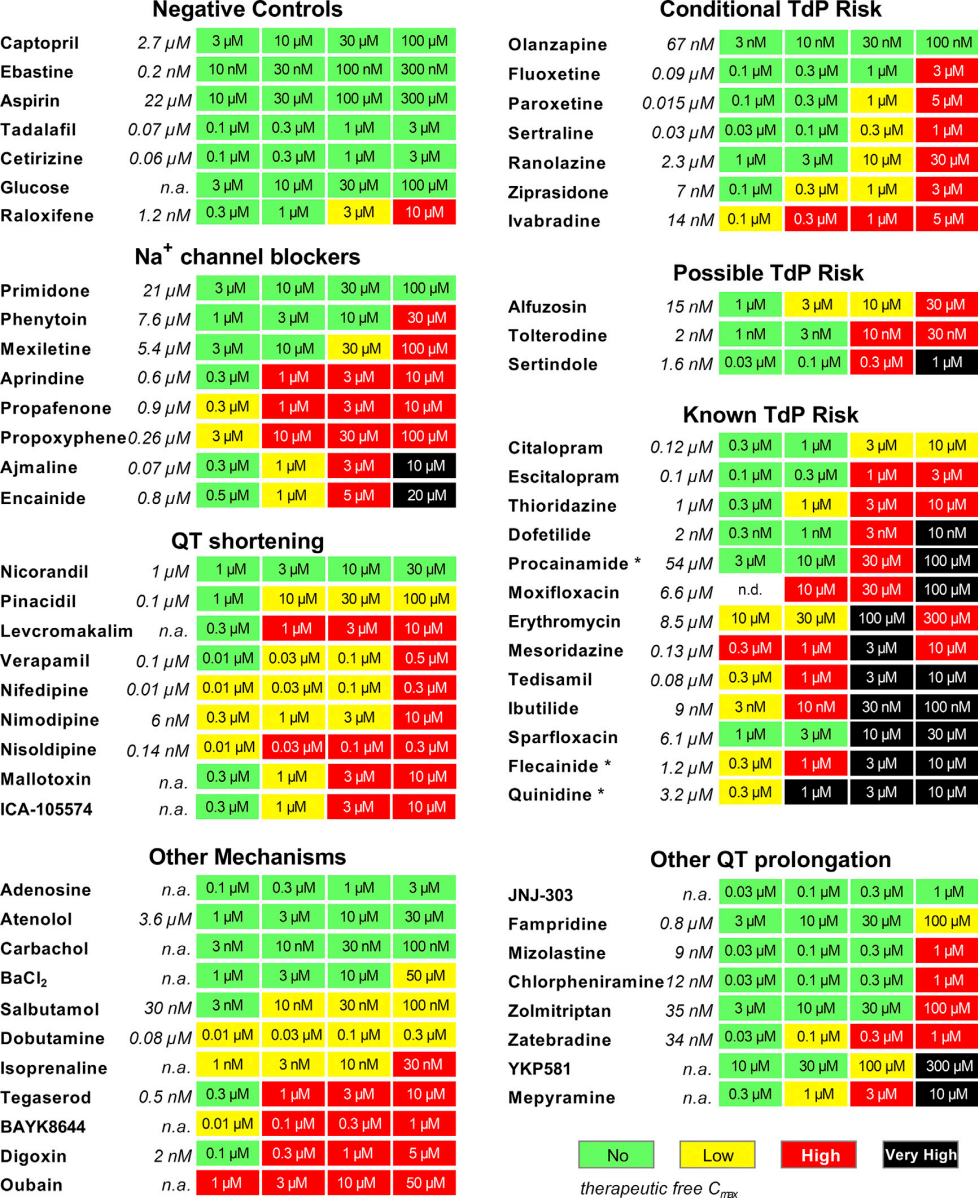
Cardiac Hazard Scoring of Reference Drugs Conditional TdP, Possible TdP, and Known TdP groups represent drugs that are listed on CredibleMeds. Concentrations were selected based on the therapeutic free C_max_ (shown in italic). ^∗^Drugs that can also be categorized as Na^+^ channel blockers. n.d., not determined; n.a., not available.

**Figure 6:**
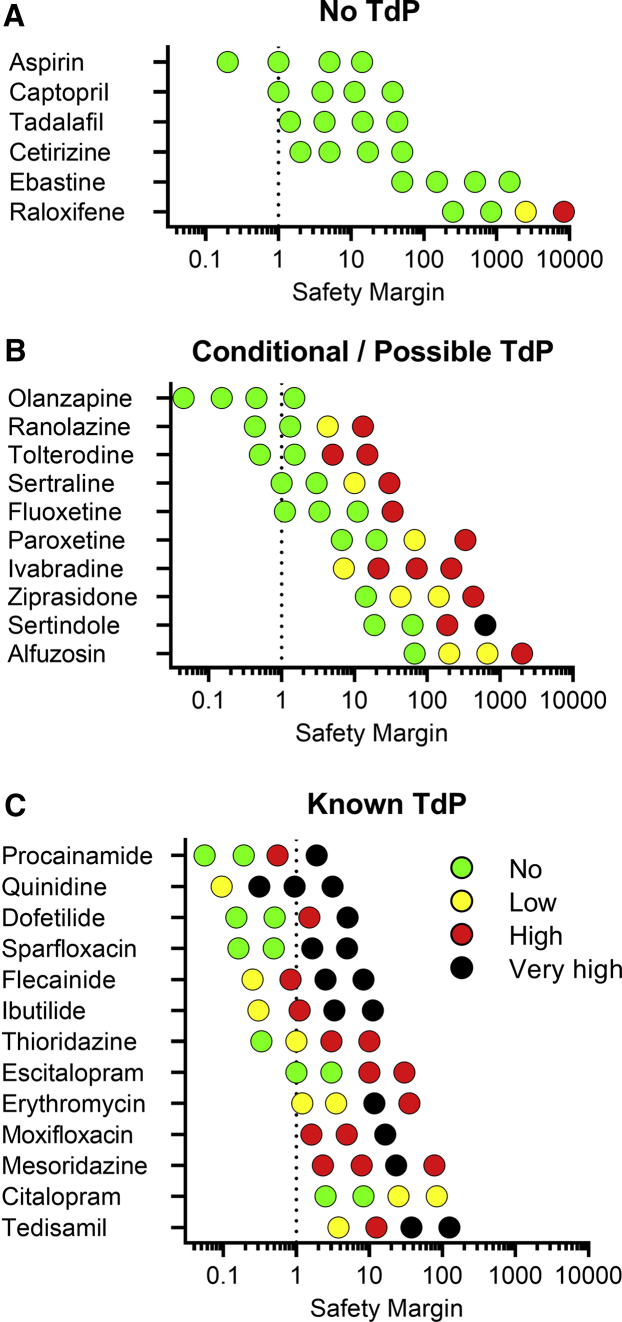
Relationship between Hazard Scoring and the Clinical Safety Margin for TdP Risk (A–C) (A) No TdP, (B) conditional and possible TdP, and (C) known TdP risk compounds plotted in function of their safety margin (tested concentration/free C_max_). Dotted line reflects the free C_max_.

Subsequently, we validated the hazard scoring (as defined in Figure 4) on the 66 reference drugs. These were grouped according to their pharmacological mechanism or level of cardiac risk in the clinic (Figure 5). Concentrations were selected to cover the therapeutic free C_max_, if applicable. Negative control drugs do not have reports of cardiac liabilities in humans and are expected to be identified as no hazard for concentrations up to 10- to 30-fold the free C_max_. Indeed, all negative controls were scored as no hazard (green). The only exception was raloxifene (3–10 μM) at clinically non-relevant concentrations, approximately ≥2500-fold the free C_max_ (Figure 6).

Next, we validated the hazard scoring system for drugs that are associated with QT prolongation and a certain degree of TdP risk in the clinic (Figures 5 and 6). Based on the CredibleMeds classification list, these drugs were categorized into three groups: (1) known risk of TdP, (2) possible (theoretical) TdP risk to long QT patients and known to cause QT prolongation, and (3) conditional TdP risk in conditions such as overdose or drug-drug interactions or certain high-risk individuals (Woosley et al., 2018). Most drugs with conditional TdP risk were scored as high hazard at concentrations 10- to 100-fold their free C_max_. Only olanzapine was not identified with any hazard, most likely since the highest tested concentration (100 nM) is around the free C_max_. Interestingly, for the conditional TdP drugs, we did not observe any EAD-like arrhythmia (very high hazard). Drugs with possible TdP risk were also identified as high hazard, but with therapeutic ratios similar to those observed with conditional TdP drugs. Sertindole was scored as very high risk at a relatively high concentration (1 μM), approximately 625-fold the free C_max_. Hence, although conditional and possible TdP drugs were identified as high hazard, arrhythmic events were relatively absent. On the other hand, 77% of known TdP risk drugs were identified as very high hazard. Arrhythmic events were detected between 0.3- and 30-fold the free C_max_ (Figure 6), which clearly indicates a very narrow safety margin for the known TdP drugs. Escitalopram and thioridazine were also identified as high hazard at 3- to 10-fold ratio, whereas citalopram was identified as only low hazard. Other drugs likely associated with a certain degree of QT prolongation (other QT prolongation group) were also identified as low to very high hazard, except for the I_Ks_ blocker JNJ-303.

Although the greatest focus within preclinical safety pharmacology is on QT prolongation, different pharmacological actions on cardiac ionic currents can also result in, e.g., QRS widening or QT shortening. The main effects observed with sodium blockers were a decrease in BR and incidence of beating arrest, which can indeed be explained by I_Na_ block causing decreased BR or complete arrest of action potential propagation in hiPSC-CMs. Such effects are then translated to low or high hazard scores as noted with, for example, phenytoin or mexiletine. Furthermore, most sodium channel blockers also have hERG-inhibiting effects in similar IC_50_ ranges, which then can be readily detected due to CTD_90_ prolongation. Aprindine, propafenone, and propoxyphene show such hazard scoring patterns, whereas encainide, quinidine, and ajmaline caused arrhythmic events. Pinacidil and levcromakalim, two cardiac I_KATP_ channel openers, both caused CTD_90_ shortening in hiPSC-CMs, which allowed identification as potentially hazardous compounds. Nicorandil is a more selective vascular I_KATP_ channel opener and was not shown to have any potential hazard risk in hiPSC-CMs, as expected. The hERG activators ICA-105574 and mallotoxin also caused CTD_90_ shortening, leading to hazard identification.

Calcium channel antagonists evoked strong responses in hiPSC-CMs, showing marked decrease in Amp, together with CTD_90_ shortening and pronounced BR increase. Since the pharmacological activity of this class of compounds is readily identified, optimization of weighted points (through an iterative approach with reference drugs) was required to minimize over-sensitization of hazard scoring to this activity. Additional mechanisms of pharmacological action were evaluated to broaden the validation exercise. Beta-adrenergic agonists (isoprenaline, dobutamine, and salbutamol) caused strong BR increase and were mainly identified as low or high hazard risk. A high hazard risk was also identified for Na^+^/K^+^ ATPase inhibitors (digoxin and ouabain), calcium channel activator BAYK8644, and tegaserod, a non-selective 5-HT_4_ agonist withdrawn from the market due to adverse cardiovascular events. Adenosine, atenolol, and carbachol were not identified for any potential hazard in the CTCM assay.

### Evaluation of the Hazard Potential of NCEs

**Figure 7:**
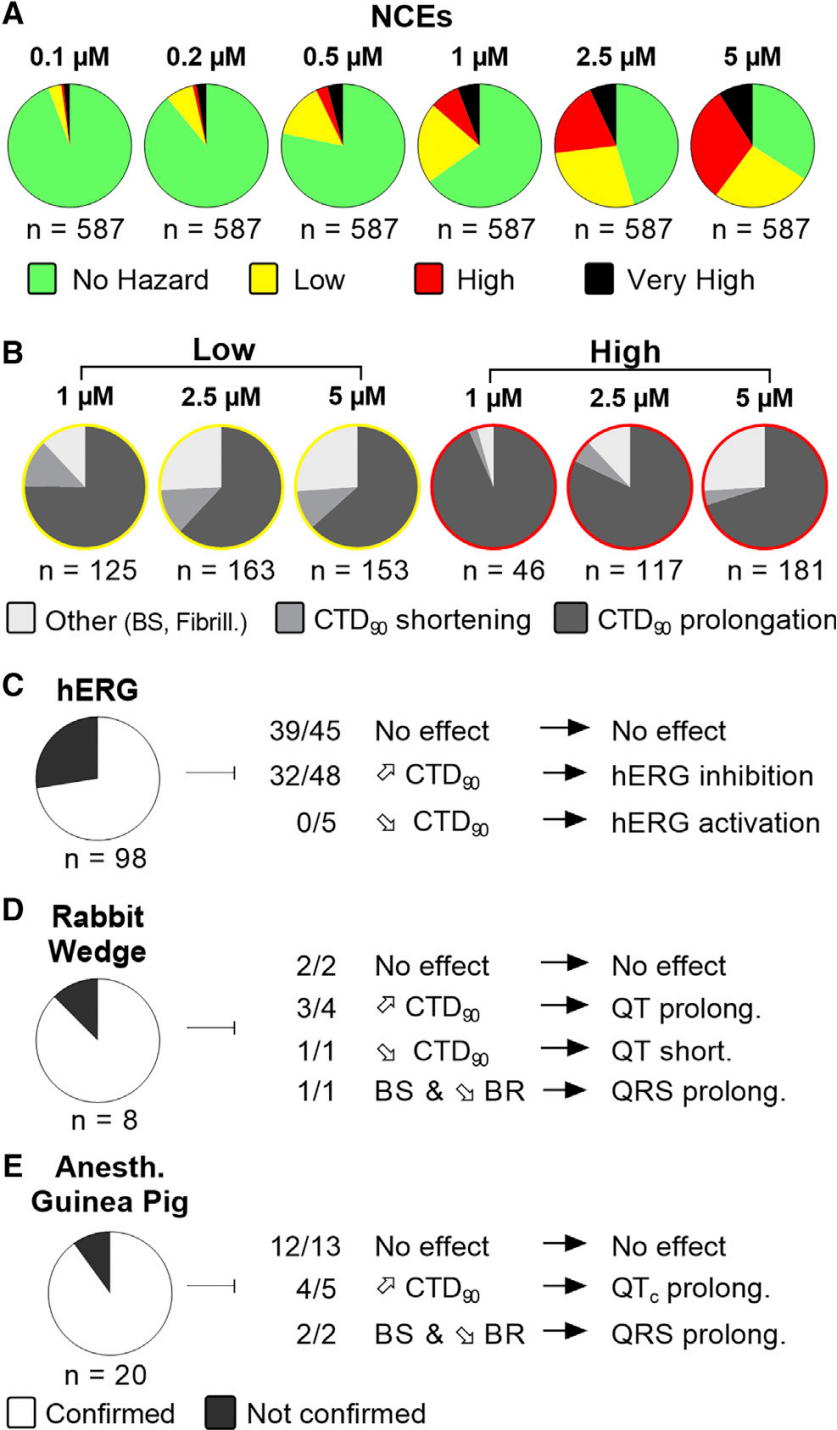
Hazard Evaluation Using the Scoring System on Janssen's NCEs in Discovery (A) Pie charts showing the concentration-dependent distribution of different hazard levels for NCEs (n = 587). (B) Analysis of CTD_90_ directional effect for NCEs scored with a low or high hazard at 1, 2.5, and 5 μM. (C–E) Translational confirmation of the CTCM-derived cardiac hazard scoring of NCEs that were also evaluated in (C) hERG, (D) isolated rabbit wedge, and (E) anesthetized guinea pig models. n indicates the number of NCEs tested in both the CTCM assay and the respective model.

Following the development and validation of the hazard scoring system, we then evaluated 587 NCEs from current Janssen discovery research programs (Figure 7A). Compounds were tested using a fixed concentration range (0.1–5 μM) that covers the therapeutic free plasma concentrations of most marketed drugs. Hazard evaluation identified that the majority of NCEs were classified within the no hazard group, except for the highest tested concentration of 5 μM. One hundred forty-eight NCEs (25%) were identified as no hazard over the entire tested concentration range. Furthermore, there was a concentration-dependent increase of all hazard levels. At 1 μM and higher, at least 33% of the compounds did show effects associated with a certain hazard level in hiPSC-CMs, with the high hazard group showing the largest contribution at 5 μM. The very high group, linked to arrhythmic-like EADs, is relatively small but with some incidences also noticed at lower concentrations.

Although the hazard classification is a first overall indicator for cardiac safety evaluation, further in-depth analysis in other models is important to get a better understanding of the respective pharmacological mechanisms leading to an integrated hazard identification. Therefore, we dissected the low and high hazard groups (at 1, 2.5, and 5 μM) with respect to the directional effect on CTD_90_ (Figure 7B). CTD_90_ prolongation seemed to be the largest cause of hazard labeling for all hazard-concentration combinations (Figure 7B). At higher concentrations, hazard identification was based on characteristics other than CTD_90_. Low hazard labeling was mainly related to beat stop incidence (<80%) and a decrease in BR and Amp (Figure S6A). High hazard labeling was related to 100% beat stop or fibrillation-like events (Figure S6B).

Finally, we evaluated the translational predictability of scored NCEs that have been also tested in our complementary cardiac safety models. Figures 7C–7E show the evaluation of CTCM findings against the hERG assay and the isolated rabbit wedge (*ex vivo*) and anesthetized guinea pig (*in vivo*) models. Changes in relevant CTCM parameters were evaluated against bidirectional percentage change in the I_Kr_ (hERG) current and (corrected) QT and QRS changes in the animal models, using overlapping concentration and/or free C_max_ ranges. The lowest confirmation rate (72%) was found with the hERG model, showing false positive and false negative signals in hERG compared with CTCM. On the other hand, the isolated rabbit wedge (88%) and anesthetized guinea pig (90%) models showed relatively high conformation rates.

## Discussion

We have presented the development of a hazard scoring system for preclinical cardiac safety evaluation of NCEs using a hiPSC-CM-based calcium transient screening (CTCM) assay. Ultimately, the application of a visual labeling and ranking of concentration-dependent compound hazard scores allowed us to simplify the interpretation of drug-induced effects on multiple parameters measured from hiPSC-CMs and to apply this approach to select cardiac-safe NCEs in early drug safety de-risking.

Our data show that the CTCM assay can identify drugs targeting various cardiac ion channels and receptors, as reported by previous studies (Bedut et al., 2016, Dempsey et al., 2016, Lu et al., 2015, Rast et al., 2015, Watanabe et al., 2017, Zeng et al., 2016). Statistical analysis of TIs in vehicle and control drug responses enabled the development of a detailed scoring matrix with differentiation of size and direction of effect per parameter. Here, we showed the value of accounting for multiple parameters (CTD_90_, BR, Amp, beat arrest, fibrillation, and EADs) in spontaneously beating hiPSC-CMs, allowing hazard identification and differentiation of various pharmacological classes of drugs. This is a more comprehensive approach compared with most other studies in hiPSC-CMs, where the focus is set exclusively on APD-related parameters (e.g., CTD_90_) associated with changes in QT interval. Therefore, our scoring system using the CTCM assay can be applied for cardiac hazard assessment beyond drug-induced QT prolongation, such as QT shortening and conduction slowing.

Nevertheless, drug-induced QT prolongation and proarrhythmic risk (e.g., TdP) remain the largest concerns within cardiovascular safety. We used the CredibleMeds classification of proarrhythmic risk of marketed drugs with evidence of certain degrees of potential TdP risk (Woosley et al., 2018). The hazard identification system could detect torsadogenic (EAD) risk for most of the high-TdP-risk drugs within the 30-fold free C_max_ range. Drugs classified as possible or conditional TdP risk were identified as high hazard or with EADs at much higher free C_max_ ratio. However, studies with dofetilide showed that EAD-like events are not always manifested in hiPSC-CMs, where strong CTD_90_ prolongation sometimes does not cause arrhythmogenic events. We believe that such variability in EAD incidence can be due to the experimental variability and batch-to-batch differences in hiPSC-CMs. However, another more likely reason is the natural complexity of EAD manifestations. Indeed, as an effort to explore the incidence of drug-associated arrhythmias in humans, a clinical pilot study reported an annual incidence of TdP of 4 in 100,000 (Darpö, 2001). This emphasizes the variable incidence of TdP in humans and the challenge to predict TdP in preclinical models. On the other hand, we have not observed any EAD-like event in over 5,000 experiments (wells) treated with negative controls (vehicle and cetirizine), suggesting that EAD-like arrhythmic behavior in hiPSC-CMs can be used as a specific surrogate for TdP. A similar outcome for proarrhythmic prediction in hiPSC-CMs has been shown using a TdP score to assess drugs with varying clinical torsadogenic risk (Ando et al., 2017). Interestingly, drug-induced incidence of EADs in hiPSC-CMs is much higher than the clinical incidence of TdP in humans, which is relatively rare (Tisdale, 2016). Hence, the CTCM assay is more sensitive for TdP risk detection, which is actually preferred for early cardiac safety de-risking in the drug discovery phase. Currently, there is an ongoing effort by pharmaceutical, regulatory, and academic scientists to evaluate the adoption of CiPA, a new preclinical paradigm for assessment of clinical risk for TdP (Colatsky et al., 2016) whereby hiPSC-CM assays are validated for detection of proarrhythmogenic potential (Millard et al., 2018, Nozaki et al., 2017) together with ion channel and *in silico* approaches. Overall, hiPSC-CMs are a valuable model to evaluate proarrhythmic risk within drug development, but careful considerations are recommended when attempting to differentiate TdP risk from apparently less concerning QT prolongation effect of drugs.

In addition to torsadogenic risk, other cardiac liabilities such as bradycardia, QT shortening, ventricular tachycardia, and fibrillation can also impede the development of a drug (Lu et al., 2008, Lu et al., 2010). These liabilities can be caused by different pharmacological mechanisms. Therefore, the scoring system was developed and evaluated to identify drugs also acting on, e.g., cardiac I_Na_, I_Ca_, I_KATP_, I_f_, Na^+^/K^+^ ATPases, and adrenergic receptors. Combination of a versatile human-based high-throughput approach and a comprehensive hazard identification tool allows an early strategic positioning of the CTCM assay within the cardiovascular safety screening paradigm. This should increase the throughput of compounds compared with the lower throughput and time-consuming *ex vivo* animal action potential models and yield more human-relevant and comprehensive data on cardiac (electrophysiological) liabilities compared with binding assays or single ion channel affinities.

Importantly, the hazard scoring system generates concentration-dependent hazard labeling rather than an overall labeling per compound. The rationale is that the “apparent” clinical (therapeutic) drug concentrations are uncertain during the different phases of preclinical drug development, and therefore it is important to score the NCEs at the level of individual concentrations. Furthermore, our approach also indicates concentration dependency and severity of hazard, facilitating the comparison and decision-making among a larger set of compounds within a specific drug discovery project. The decision-making is also dependent on the therapeutic benefit-risk (e.g., life-threatening versus non-life-threatening less serious diseases), which can influence the interpretation of various hazard labels. Hence, we defined the color label for very high hazard as black, since this indicates potential life-threatening TdP risk due to observed EAD-like events. High hazard (red label) profiles of lead compounds should be assessed in more detail and further investigated with follow-up cardiovascular safety studies. Evaluation of a large sampling of our internal NCEs from across drug discovery projects clearly showed a concentration-dependent increase in different hazards. A more in-depth analysis indicated that both low and high hazard labels were predominantly associated with CTD_90_-prolonging effects. This might be related to previous findings showing that many NCEs display hERG-inhibiting effects around 5 μM (Lu et al., 2008). Validation of the hazard scoring in CTCM against our other cardiac safety models showed that the translational confirmation of CTCM findings was relatively high in the isolated rabbit wedge and anesthetized guinea pig models, indicating good predictivity of the CTCM-based hazard scoring system. Since hiPSC-CMs represent a more complex cardiac model than only I_Kr_, the translation to the hERG assay was, as expected, lower compared with *in/ex vivo* models.

The scoring system can be applied not only to various calcium-imaging-based assays but also to different detection technologies and experimental models. Using the same principal (i.e., design of the scoring matrix and the hazard labeling), the scoring system could be developed for technologies based on multi-electrode arrays (Yamazaki et al., 2018), voltage-sensitive dyes (Blinova et al., 2017, Lu et al., 2017), impedance (Zhang et al., 2016), and video motion imaging (Kopljar et al., 2017) to comprehensively account (indirectly) for APD-like parameters as well as other pharmacological responses such as BR, beat stop, and EAD-like events. Similarly, different cardiomyocyte models (primary or stem cell-derived) can be used. Furthermore, studies have shown the potential of machine learning approaches in cardiac pharmacology classification (Heylman et al., 2015, Lee et al., 2017). Such approaches might refine the scoring matrix to further optimize the hazard scoring system.

Although the hazard scoring system is a promising tool, we want to emphasize the limitations of our approach. Drug responses possess a certain degree of variability. In our model, this means that variability in (Δ)Δ% effects together with the use of static cutoffs can eventually lead to different hazard labeling (between no/low and low/high). Limitations of hiPSC-CMs and applied technologies should also be taken into consideration. hiPSC-CMs represent a fetal-like phenotype, with a more immature morphology, force contractility, calcium handling, and energy metabolism compared with adult native cardiomyocytes (Yang et al., 2014). With respect to cardiac electrophysiology, calcium channel antagonists evoke strong responses on the BR of hiPSC-CMs, which needs to be considered within the hazard system. On the other hand, I_Ks_ blockers (e.g., JNJ-303) cannot be easily detected in *ex vivo* primary myocytes (Varró et al., 2000) or in hiPSC-CMs, most likely due to the role of I_Ks_ as a repolarization reserve (Braam et al., 2013) and the lack of adrenergic activation *in vitro*. Sodium channel blockers can be detected but not easily differentiated from other pharmacological mechanisms. Calcium transient imaging is an invasive approach in which the calcium dyes can affect the physiology of the model (Bootman et al., 2018), although we have optimized the CTCM assay to minimalize such impacts (Kopljar et al., 2018). In addition, we recommend timely re-analysis of TIs and experimental variability to account for possible changes in hiPSC-CM cultures or experimental procedures over time. Furthermore, statistical analysis (TIs) should be used only as a supportive tool and not an arbitrary approach for the determination of cutoffs. Finally, we have not examined the application of the hazard scoring on other cell lines. Different hiPSC-CM cell lines can possess experimental and phenotypic variability (Sala et al., 2017), although a cross-site validation study showed that drug categorization for TdP risk was comparable between two commercial cell lines (Blinova et al., 2018).

In summary, the development of a cardiac scoring system using calcium imaging in hiPSC-CMs allows early preclinical hazard identification for NCEs in a high-throughput modality. The system allows a more comprehensive identification of multiple pharmacological actions affecting cardiac electrophysiology in hiPSC-CMs using a composite score and can identify different levels of TdP risk. Furthermore, the methodological approach for devising the hazard scoring system could be applied to other cardiac-related assays as well as other biomedical screening assays for risk quantification.

## Experimental Procedures

The *in vivo*/*ex vivo* data analysis in this study originates from animal care and experimental procedures conducted at Janssen Pharmaceutica facilities in accordance with the European Directive of September 22, 2010, regarding the protection of animals that are used for scientific purposes and the Belgian Act of May 29, 2013 on the implication of this directive.

### Cell Culture and Reagents

hiPSC-CMs were purchased from Ncardia (Cor.4U cardiomyocytes, Ax-C-HC02-96) as living pre-plated cells seeded onto fibronectin-coated 96-well μClear plates (Greiner Bio-One, No. 655090) at a density (∼25,000 cells/well) suited to forming a confluent synchronously beating monolayer. Cor.4U cardiomyocytes represent a mix of 60% ventricular, 30% atrial, and 10% nodal cells according to the cell provider. Cells were cultured with Cor.4U culture medium (Ax-M-HC250) in a humidified incubator at 37°C and 5% CO_2_, with medium being changed once a day. On the day of the experiment, the culture medium was replaced with Tyrode's solution (Sigma, No. T2397) supplemented with 10 mM HEPES together with KCl to represent isokalemic (4.2 mM K^+^) conditions.

Table S1 contains the purchase information and free C_max_ references for all the compounds used within this work. The preparation of drug solutions is explained in Supplemental Experimental Procedures. The calcium-sensitive fluorescence dye Cal-520 AM (Cat. No. 36,338; AAT Bioquest) was used to capture the intracellular calcium transients in hiPSC-CMs. The protocol was used as described in Kopljar et al. (2018). Briefly, Cal-520 was incubated for 45 min followed by a washout and a 30-min recovery before starting the experiments.

### Calcium Transient Measurements

The spontaneous beating activity of hiPSC-CMs was assessed through measurement of the Ca^2+^ fluorescence signal integrated over the whole well. Fluorescence signals were measured using the FDSS/μCell platform and the records subsequently analyzed offline using NOTOCORD-hem software (version 4.3), containing EXT modules and an algorithm developed by XiTechniX to detect beat-by-beat Amp, BR, and CTD_90_ parameters (Figure S7). All wells within a plate were measured simultaneously using the following FDSS/μCell settings: sampling frequency 66.7 Hz, exposure time 14.6 ms, excitation wavelength 480 nm, emission wavelength 540 nm, temperature controlled at 37°C.

First, the experimental plates were put into the FDSS/μCell to stabilize for 10 min. Next, a baseline recording was run for 3 min followed by compound addition. The effect of a compound was recorded (5-min recording time) around 15 and 30 min after compound addition. CTD_90_, BR, and Amp were quantified for baseline and 30-min compound effects as the median value of all beats (calcium transients) measured within a 1-min interval of the recording. The recording around 15 min was used only for observation of EADs or fibrillation-like events. EADs were manually monitored and evaluated. Beat stop was defined after 30 min in case BR was <5 beats/min. Wells that temporarily stopped beating during compound addition but recovered at the 30-min time point were not defined as beat stop.

### Quality Control

Wells showing no beating or non-synchronous beating at baseline were excluded from analysis. Wells with a BR at baseline lower than 30 or higher than 90 beats/min were also excluded. Plates with more than 10% of the wells outside of the BR criteria were entirely excluded. Further quality control of the plates was evaluated using positive control drugs. Plates were excluded under the following criteria: (1) <15 ΔΔ% change on CTD_90_ with dofetilide at 3 nM, (2) <30 ΔΔ% change on BR with isoprenaline at 0.1 μM, and (3) >−10 ΔΔ% change on CTD_90_ with nimodipine at 0.1 μM. In total, 13/256 (5.1%) plates were excluded, 8 plates because of BR criteria and 5 plates because of positive control criteria.

### Statistical Analysis

The responses were measured at baseline and at 30 min. Consequently, the Δ% effect was calculated for each experiment and each response using the following formula (example of CTD_90_):

$$CTD_{90}\Delta \%=100\cdot \frac{CTD_{90}\left(30\;mins\right)-CTD_{90}\left(baseline\right)}{CTD_{90}\left(baseline\right)}\text{.}$$

Hence, Δ% effect reflects changes at 30 min with respect to the baseline values. To avoid possible influence of plate effect, further adjustment of the data was performed by calculation of ΔΔ% (net) effect, which adjusts the drug effects based on effects observed for vehicle in a given plate. The drug and vehicle effect is represented as the median of the individual experiments observed within the respective plate (n = 4–8 per plate). Hence:

$$CTD_{90}\Delta \Delta \%=\;CTD_{90}\Delta \%\;\left(drug\right)-CTD_{90}\Delta \%\;\left(vehicle\right)\text{.}$$

Non-parametric TIs were calculated with Wilks' approach at 95% confidence level covering 90% of a population (more details are provided in Supplemental Information).

## Author Contributions

I.K., H.L., A.T., and D.J.G. conceived the study. I.K. performed and analyzed the calcium transient assay experiments. M.O. and F.T. designed and conducted statistical analysis. I.K. and H.L. developed the hazard scoring system, with input from K.V.A., A.T., and D.J.G. K.V.A. performed the hazard scoring analysis. I.K. wrote the paper. All authors reviewed the manuscript and approved the final version.

## Supplemental Information

Document S1. Supplemental Experimental Procedures, Figures S1–S7, and Table S1

Document S2. Article plus Supplemental Information
