# Supplementary material for: Development of a Human iPSC Cardiomyocyte-Based Scoring System for Cardiac Hazard Identification in Early Drug Safety De-risking
Source: Stem Cell Reports. 2018 Dec 11;11(6):1365–77. doi: 10.1016/j.stemcr.2018.11.007 (PMC6294263; doi:10.1016/j.stemcr.2018.11.007)
Supplement: Document S1. Supplemental Experimental Procedures, Figures S1–S7, and Table S1 [file mmc1.pdf]

Stem Cell Reports, Volume *11*

## Supplemental Information

# Development of a Human iPSC Cardiomyocyte-Based Scoring System for Cardiac Hazard Identification in Early Drug Safety De-risking

**Ivan Kopljar, Hua Rong Lu, Karel Van Ammel, Martin Otava, Fetene Tekle, Ard Teisman, and David J. Gallacher**

**Supplemental Figures**

**Figure S1**

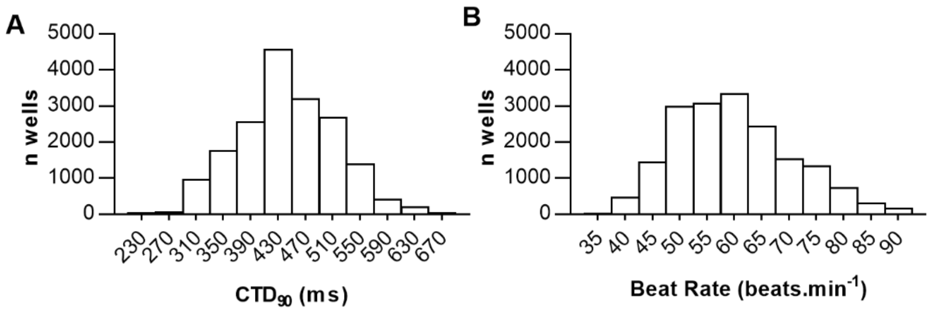


**Figure S1**. **Beating properties of hiPSC-CMs. Related to Figure 2.** Histograms reflecting a Gaussian distribution of baseline beating properties ($CTD_{90}$ and BR). Data is based on n = 23,183 independent experiments used within the entire analysis that have passed the quality control criteria.

**Figure S2**

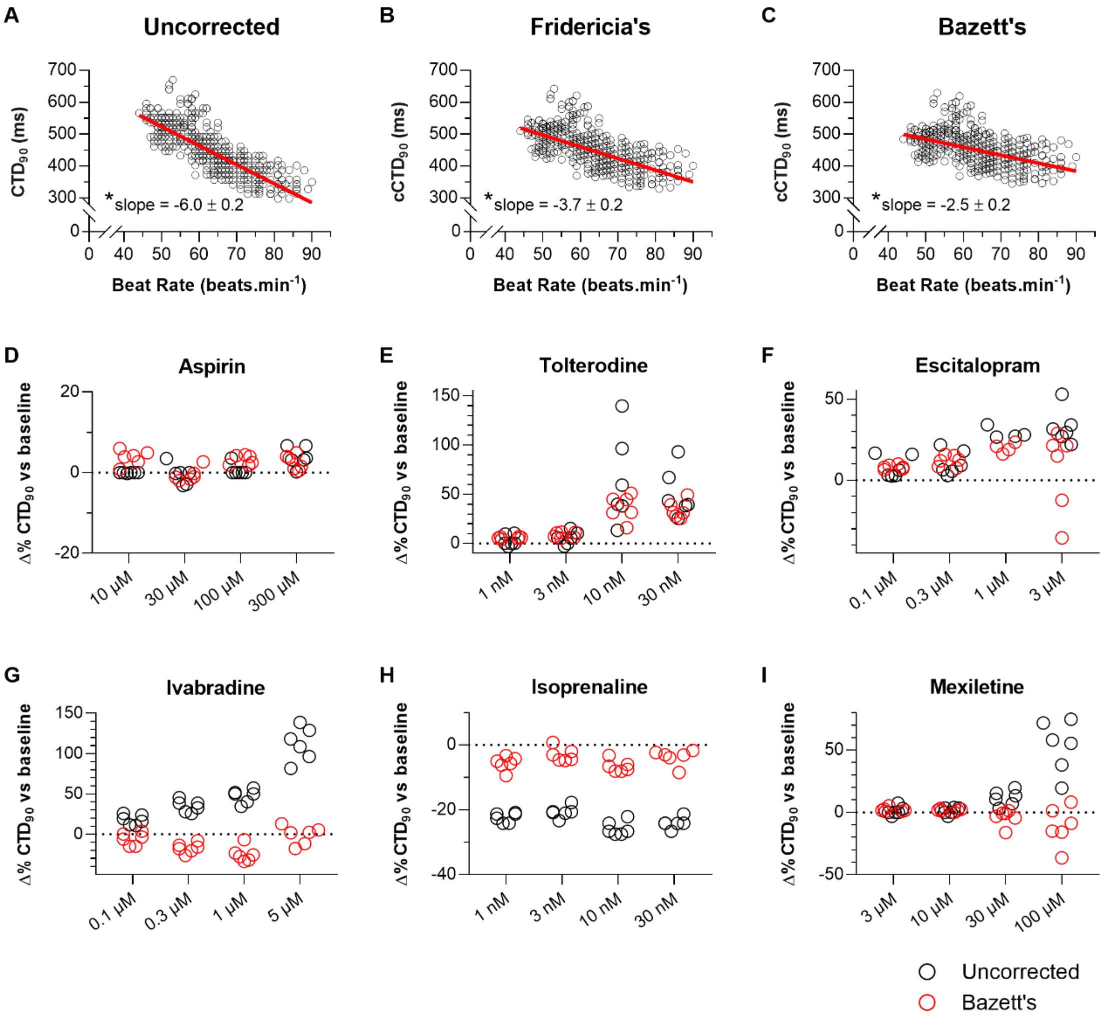


**Figure S2**. **Correction of $CTD_{90}$ in spontaneously beating hiPS-CMs. Related to Figure 2.** A) Relationship between $CTD_{90}$ and BR using uncorrected baseline experiments, fitted with a linear regression (red line). Correction of $CTD_{90}$ values from A) using B) Fridericia's and C) Bazett's correction formulas still displays a correlation between corrected (c)$CTD_{90}$ and BR. n = 728. Slope is represented as best-fit value ± S.E.M. *: significant deviation from zero; p<0001. D-I) Examples of drug-related Δ% changes for Bazett-corrected compared to uncorrected $CTD_{90}$ values. n = 6 independent experiments. Note for certain drugs strong (directional) differences between uncorrected and corrected data points.

**Figure S3**

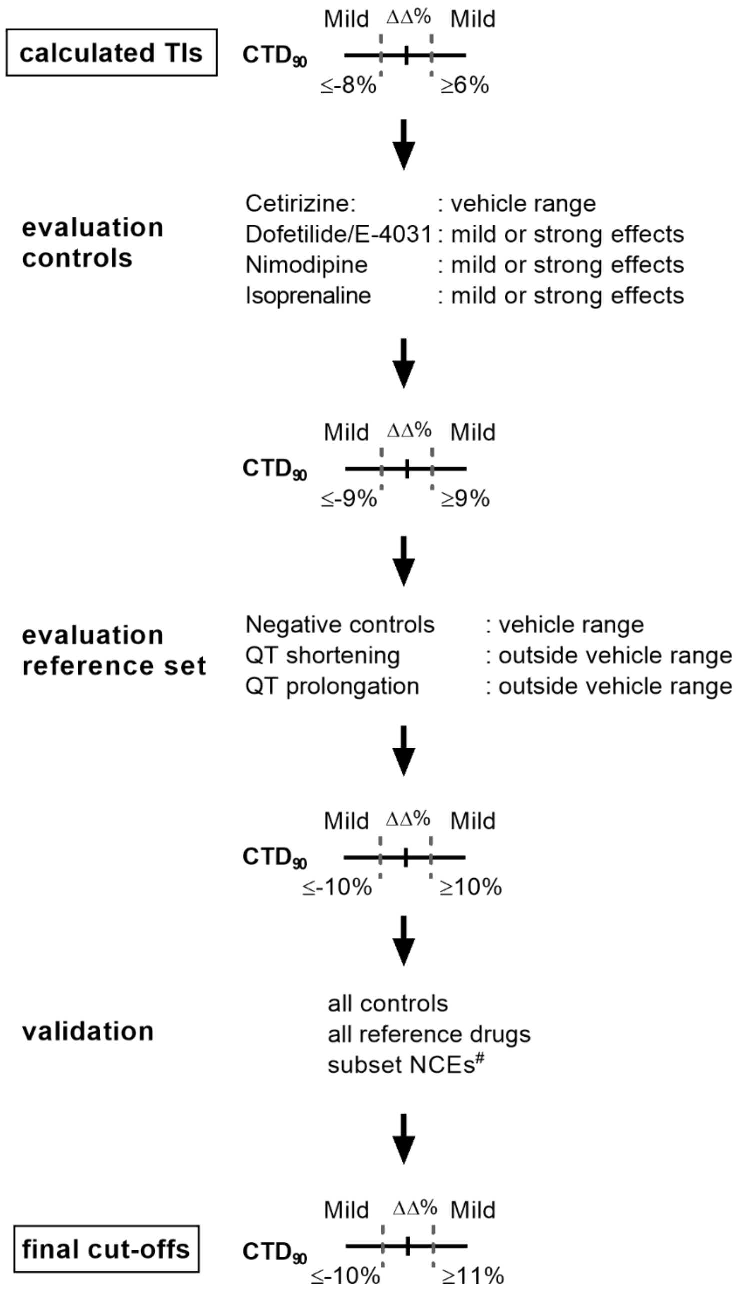


**Figure S3**. **Optimization of mild $CTD_{90}$ cut-offs. Related to Figure 4.** Process of optimization of mild changes in $CTD_{90}$ starting from calculated TIs. #A subset of NCEs was used to evaluate possible false positive hazard labeling in NCEs. Based on the validation step, the cut-off for $CTD_{90}$ prolongation was set to 11%. This was considered the cut-off where false positives are minimalized whereas the sensitivity for true positive signals is still sufficient.

**Figure S4**

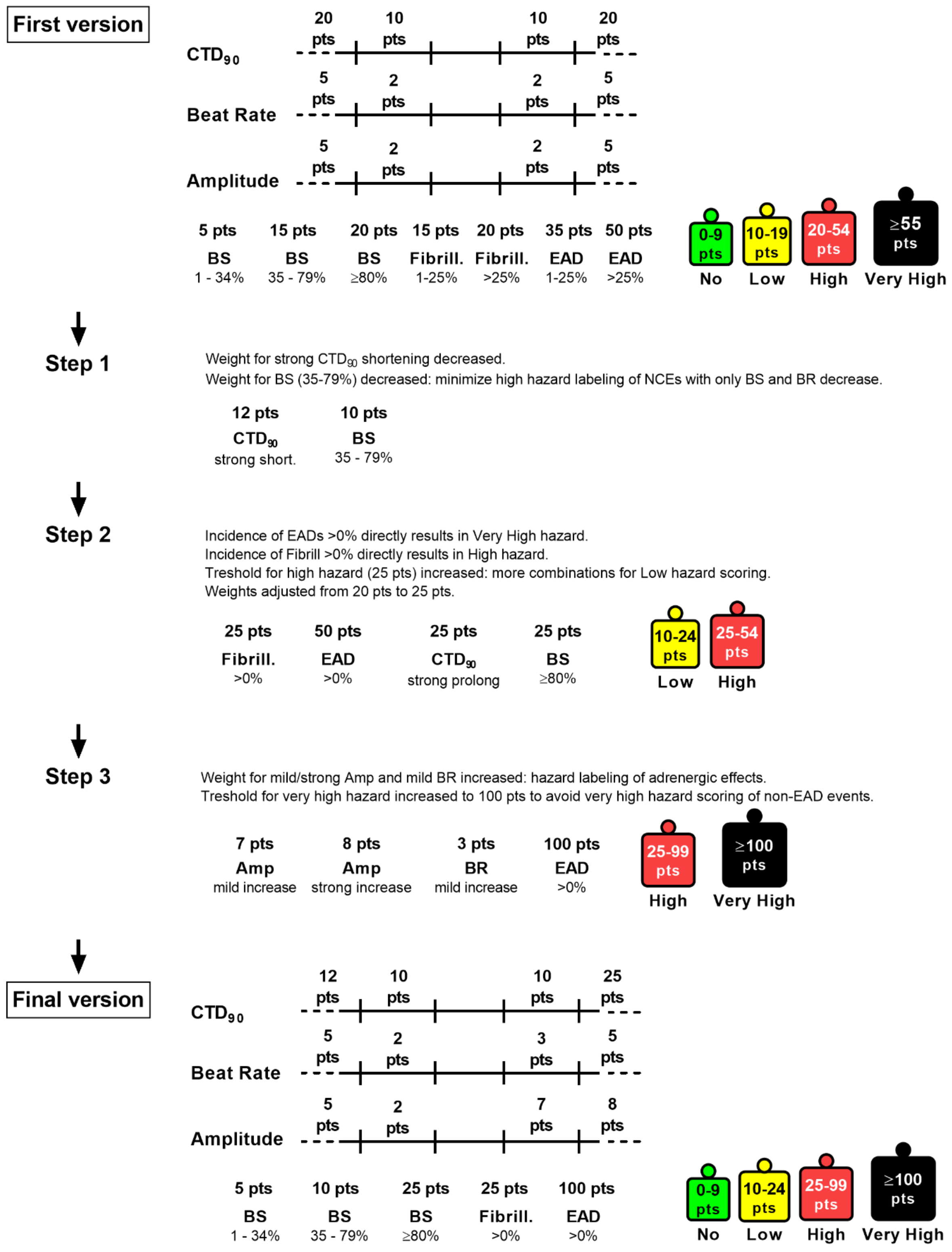


**Figure S4. Optimization of the scoring matrix. Related to Figure 4.** Process of optimization of the weights and hazard labels.

**Figure S5**

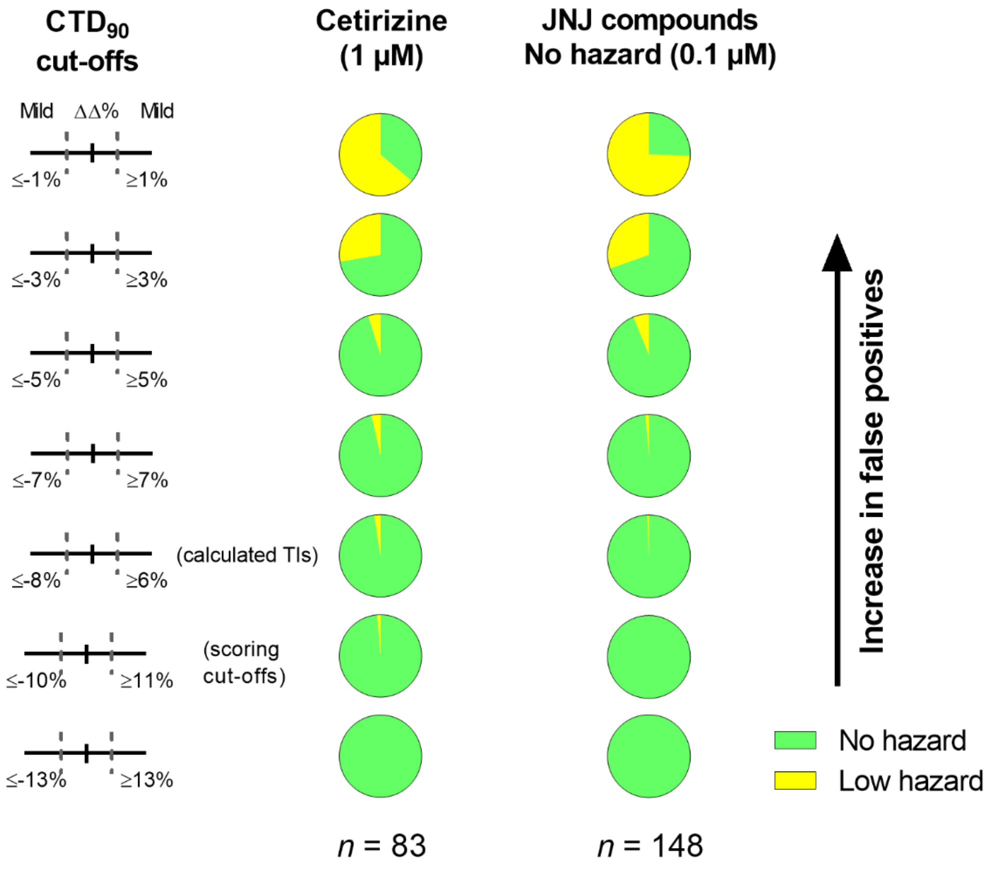


**Figure S5. The effect of $CTD_{90}$ cut-off selection on false positive hazard labeling. Related to Figure 4.** Different cut-offs shown as ΔΔ% changes were evaluated on the negative control cetirizine (1 µM) and on Janssen's NCEs (0.1 µM) which showed an overall no hazard profile within the tested concentration range (0.1–5 µM).

**Figure S6**

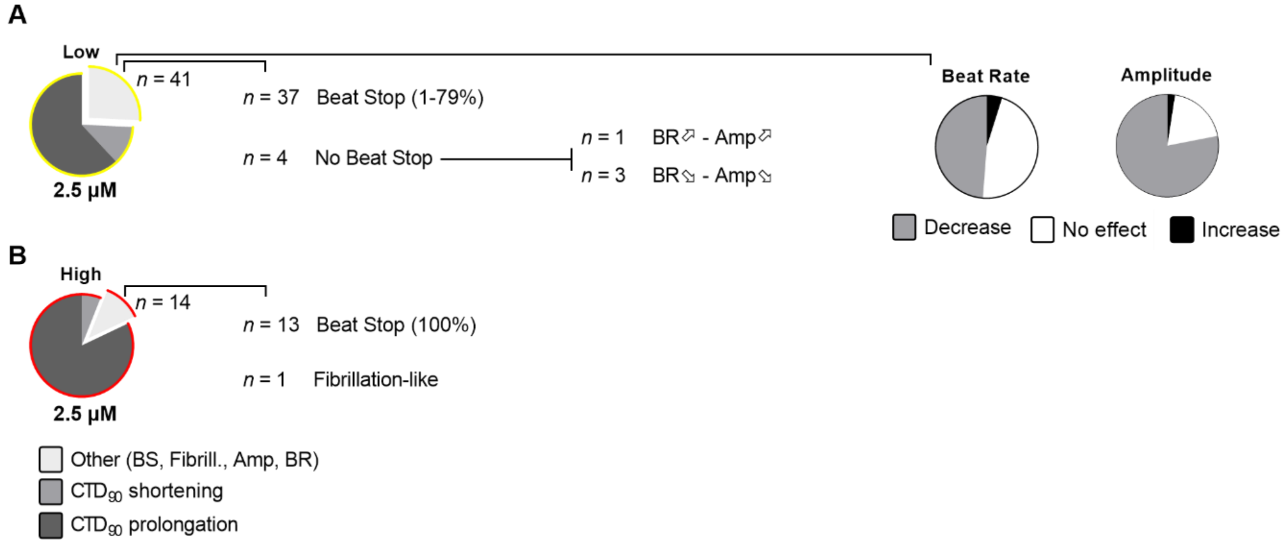


**Figure S6**. **The impact of non-CTD$_{90}$ parameters on hazard labeling of NCEs. Related to Figure 7.** Detailed analysis of A) low and B) high hazard labeling of NCEs (2.5µM) not related to CTD$_{90}$ changes. n indicates the number of evaluated NCEs.

**Figure S7**

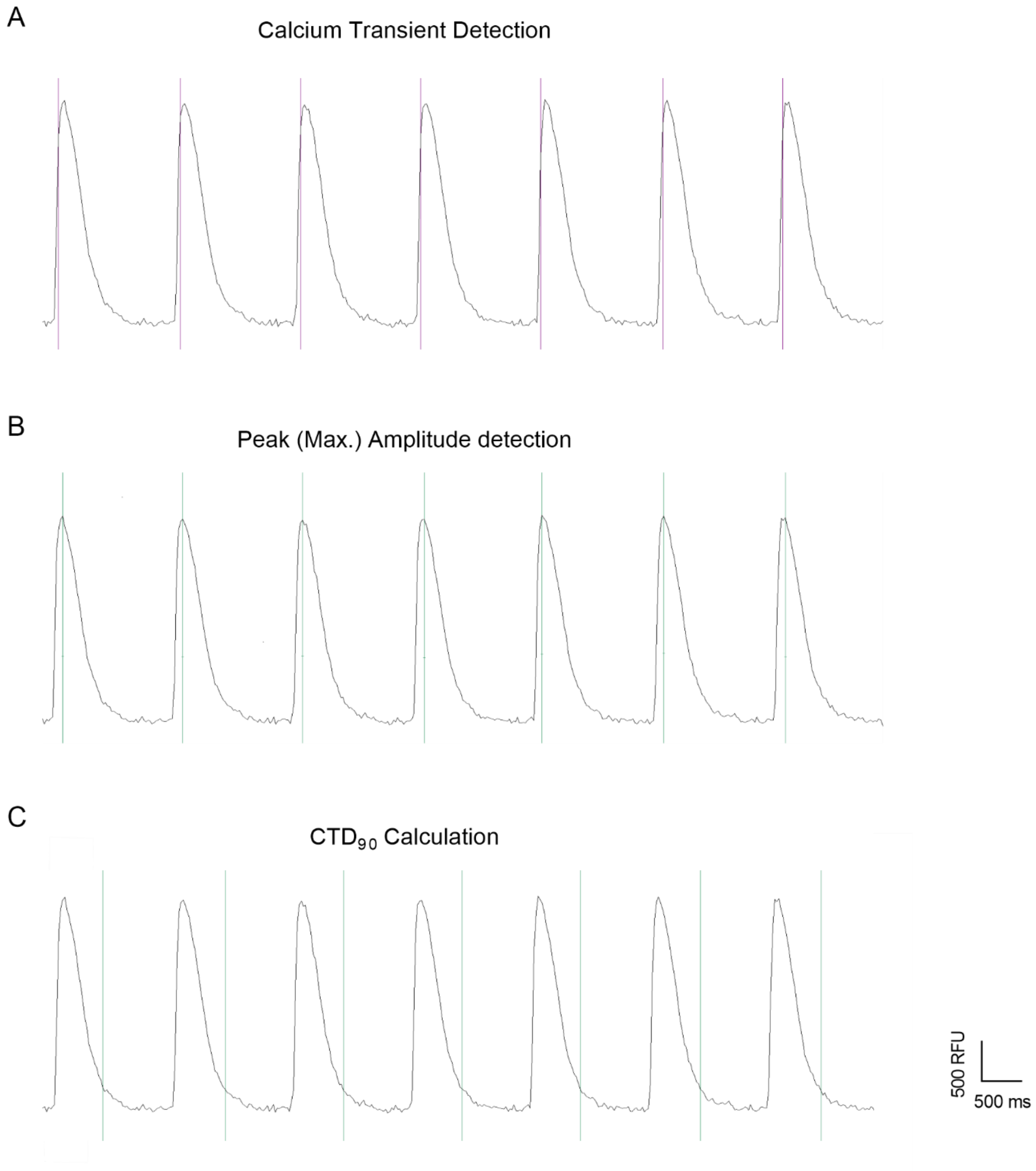


**Figure S7**. **Calcium transient detection algorithm. Related to Figure 2.** A) Beat/cycle detection. B) Peak amplitude detection. C) $CTD_{90}$ detection/calculation.

**Table S1**

| Compound | Vendor | Product nr. | Lot nr. | Reference free Cmax |
|---|---|---|---|---|
| Adenosine | Sigma-Aldrich | D9434 | SLBH3471V | not available |
| Ajmaline | Janssen Research Foundation | not available | not available | Redfern et al. (2003) |
| Alfuzosin | Sigma-Aldrich | A0232 | 072M4744V | Schulz et al. (2012) |
| Aprindine | Sigma-Aldrich | A7606 | 038K4711V | Harmer et al. (2011) |
| Aspirin | Sigma-Aldrich | A5376 | SLBN2916V | Yao et al. (2008) |
| Atenolol | Sigma-Aldrich | A7655 | BCBR3720V | Schulz et al. (2012) |
| BaCl2 | Sigma-Aldrich | 342920 | MKBH4234V | not available |
| BAYK8644 | WuXi AppTec | EO7563_3_001 | not available | not available |
| Captopril | Sigma-Aldrich | C4042 | BCBP9930V | Yao et al. (2008) |
| Carbachol | Sigma-Aldrich | PHR1511 | LRAA3318 | not available |
| Cetirizine | Sigma-Aldrich | C3618 | 122M4712V | Redfern et al. (2003) |
| Chlorpheniramine | Sigma-Aldrich | C3025 | not available | Ando et al. (2017) |
| Citalopram | Sigma-Aldrich | PHR1640 | LRAA6012 | Harmer et al. (2011) |
| Dextropropoxyphene | Janssen Research Foundation | not available | not available | Schulz et al. (2012) |
| Digoxin | Sigma-Aldrich | D6003 | 100M1327V | Schulz et al. (2012) |
| Dobutamine | Sigma-Aldrich | D0676 | 055M4018V | Banner et al. (1991) |
| Dofetilide | TOSLab Ltd. | 3979/1 | not available | Redfern et al. (2003) |
| Dofetilide | Sigma-Aldrich | PZ0016 | 030M4707V | Redfern et al. (2003) |
| Ebastine | Sigma-Aldrich | E9531 | 028K4712V | Redfern et al. (2003) |
| Encainide | Sigma-Aldrich | E9156 | 018K4611V | Harmer et al. (2011) |
| Erythromycin | Sigma-Aldrich | E5389 | WXBC1653V | Redfern et al. (2003) |
| Escitalopram | Sequoia Research Products | SRP01460e | not available | Schulz et al. (2012) |
| Fampridine | ACROS CHIMICA | 104570050 | A0303988 | Schulz et al. (2012) |
| Flecainide | WuXi AppTec | EO7972_2_002 | not available | Ando et al. (2017) |
| Fluoxetine | Sigma-Aldrich | PHR1394 | LRAA9180 | Redfern et al. (2003) |
| Glucose | Sigma-Aldrich | A9251 | SLBL0630V | not available |
| Ibutilide | Sigma-Aldrich | I9910 | 035M4776V | Redfern et al. (2003) |
| ICA-105574 | WuXi AppTec | EO7972_3_001 | not available | not available |
| Isoprenaline | TCI Chemicals | I0261 | YCMCK-JO | not available |
| Ivabradine | WuXi AppTec | EO7548_6_001 | not available | Camm (2006) |
| JNJ-303 | TOCRIS COOKSON | 3899 | 4A/179434 | not available |
| Levcromakalim | Sigma-Aldrich | P154 | BGBC4503 | not available |
| Mallotoxin | Sigma-Aldrich | V4629 | MKBV4993V | not available |
| YKP581 | SK LIFE SCIENCE | YKP581 | BP-01-01-38 | not available |
| Mepyramine | Fluka | PHR1340 | LRAA1092 | not available |
| Mesoridazine | Sigma-Aldrich | M4068 | 081M4705V | Harmer et al. (2011) |
| Mexiletine | Sigma-Aldrich | M2727 | 099K1483 | Ando et al. (2017) |
| Mizolastine | Sigma-Aldrich | CDS021588 | 1582511 | Redfern et al. (2003) |
| Moxifloxacin | Carbosynth Ltd | FM65095 | 1506016644m | Harmer et al. (2011) |
| Nicorandil | Sigma-Aldrich | R5648 | SLBL9875V | not available |
| Nifedipine | Sigma-Aldrich | N7634 | MKBR1676V | Harmer et al. (2011) |
| Nimodipine | Prestwick | Prestw-918 | not available | Schulz et al. (2012) |
| Nisoldipine | WuXi AppTec | EO7548_5_001 | not available | Schulz et al. (2012) |
| Olanzapine | Sigma-Aldrich | O1141 | 035M4781V | Harmer et al. (2011) |
| Ouabain | Sigma-Aldrich | O3125 | 021M1512V | not available |
| Paroxetine | Johnson and Johnson Pharma | not available | not available | Schulz et al. (2012) |
| Phenytoin | Sigma-Aldrich | PHR1139 | P500169 | Harmer et al. (2011) |
| Pinacidil | Sigma-Aldrich | N3539 | 051M4729V | Thuillez et al. (1991) |
| Primidone | Sigma-Aldrich | P7295 | not available | Schulz et al. (2012) |
| Procainamide | Sigma-Aldrich | P9391 | SLBG4388V | Redfern et al. (2003) |

| | | | | |
|---|---|---|---|---|
| Propafenone | Sigma-Aldrich | P4670 | MKBR4240V | Harmer et al. (2011) |
| Quinidine | Sigma-Aldrich | not available | not available | Redfern et al. (2003) |
| Raloxifene | Sigma-Aldrich | R1402 | MKBS2409V | Czock et al. (2005) |
| Ranolazine | Sigma-Aldrich | R6152 | not available | Ando et al. (2017) |
| Salbutamol | Sigma-Aldrich | S8260 | 071M1166V | Schulz et al. (2012) |
| Sertindole | Sigma-Aldrich | S8072 | BGBC4254V | Redfern et al. (2003) |
| Sertraline | TCI Chemicals | S0507 | R8VYD-EC | Harmer et al. (2011) |
| Sparfloxacin | Sigma-Aldrich | 56968 | BCBN3519V | Yao et al. (2008) |
| Tadalafil | Kemprotec | K-1117 | not available | Schulz et al. (2012) |
| Tedisamil | KALI-CHEMIE | KC8857 A5-1/M | not available | Redfern et al. (2003) |
| Tegaserod | Sigma-Aldrich | SML1504 | 016M4704V | Appel-Dingemanse et al. (2002) |
| Thioridazine | Sigma-Aldrich | T9025 | BCBQ9396V | Redfern et al. (2003) |
| Tolterodine | Sigma-Aldrich | PZ0009 | 100M4706V | Olsson et l. (2001) |
| Verapamil | WuXi AppTec | EO7972_1_001 | not available | Schulz et al. (2012) |
| Zatebradine | Sigma-Aldrich | Z0127 | 081M4613V | Roth et al. (1993) |
| Ziprasidone | USP | 1724408 | F1J028 | Ando et al. (2017) |
| Zolmitriptan | Sigma-Aldrich | SML0248 | 022M4724V | Schulz et al. (2012) |

**Table S1. Compound purchase information and free $C_{max}$ references. Related to Figure 5.**

## Supplemental Experimental Procedures

Preparation of drug solutions

Compounds were dissolved in dimethyl sulfoxide (DMSO) to obtain a stock solution of 1000-fold the highest test concentration, which was then further diluted to obtain concentrations of 1000-fold the intended concentration. On the day of experiment, these solutions were further diluted with the supplemented Tyrode's solution. Compound addition was done automatically using the Functional Drug Screen System (FDSS/µCell; Hamamatsu, Japan) head stage by adding 100 µL of the 2-fold compound solution to wells with hiPSC-CMs already containing a volume of 100 µL of the experimental solution, finally reaching the intended test concentration in 0.1% DMSO.

Calcium transient detection algorithm

The analysis algorithm consists mainly of multiple parts (beat/cycle, feature and parameter detection) that are executed sequentially. The first part is a beat/cycle detection algorithm using filtering and auto thresholding techniques. The feature detection algorithm then identifies for each detected beat/cycle the beginning (e.g. minimum) and the top (e.g. maximum) of the calcium transient. The third part is a parameter calculation algorithm which uses the detected features to calculate the amplitude and $CTD_{90}$. Beat rate is calculated based on the time interval between different calcium transient peaks (max.).

Process of defining the weighted points

Defining of the weighted points was based on certain criteria that should reflect the expected hazard labeling for certain drug classes. Here, we explain the most important criteria (requirements) that we applied to design the weighted points system through an iterative approach. The weighted algorithm was first evaluated on the control drugs (Fig. 4C), followed by validation and further fine-tuning using the 66 reference drugs. The first requirement was to have a unique labeling of tested concentrations where EADs were observed (very high hazard). As such, EADs were given 100 points, whereas the cumulation of all other combinations could never reach the 100 points minimum required for very high hazard labeling.

The next requirement was to account for drug responses which would be considered as high hazard. Strong $CTD_{90}$ prolongations (based on dofetilide and E-4031) which could potentially lead to EADs were given 25 points, which was also the minimal number of points required to receive the high hazard label. Mild $CTD_{90}$ prolongations, which also showed clear changes in BR and Amp together with a certain incidence (35-80%) of BS, were also expected to be identified as high hazard. In case all (or most) of the wells showed BS, there would be no primary parameter data available and therefore a high incidence of BS (>80%) was scored as 25 points to reflect the high hazard of this type of response. Also $CTD_{90}$ shortening in combination with changes in BR, Amp and a certain incidence of BS received high hazard labeling. Furthermore, $CTD_{90}$ shortening in combination with strong BR increase and Amp increase were weighted to receive a high hazard label, since this phenotype is observed with strong adrenergic stimulation. On the other hand, $CTD_{90}$ shortening in combination with strong BR decrease and Amp decrease were weighted to receive a low hazard labeling (unless additional BS incidence was observed), since this phenotype most likely reflects calcium antagonism, to which hiPSC-CMs are particularly sensitive. Fibrillation-like observations were also directly associated with high hazard and therefore given 25 points.

Mild $CTD_{90}$ changes in most cases were expected to be labeled as low hazard. A combination of strong changes in BR and Amp, but without any $CTD_{90}$ changes, were relatively rare but could sometimes be observed with e.g. sodium channel blockers. Therefore, strong BR and Amp changes were weighted to reach an accumulated minimum of 10 points (low hazard). Mild decreases in BR and/or Amp were not identified as a hazard. A combination of mild Amp and BR increase was considered an indication of an adrenergic stimulation and therefore labeled as low hazard.

Tolerance interval calculations

Non-parametric tolerance intervals (TIs) were calculated with Wilks' approach (Wilks, 1941) at 95% confidence level covering 90% of population (more details are provided in Supplemental Information). Non-parametric approach truncates number of the lowest and the highest observed values to obtain interval bounds. Wilks' approach utilizes beta distribution to determine number of observations to be truncated to achieve specified confidence and coverage levels. Truncation is performed symmetrically based on Wilks' approach (same number truncated for the lowest and the highest values). The calculations presented in Figure 3 were done on a subset of vehicles and control drugs (mainly plates where reference drugs were tested) based on data from individual experiments. It is important to note that for the calculation of TIs that are supposed to characterize an "usual population", the data set needs to represent the expected effects. Hence, individual experiments were excluded when they showed an unexpected response in hiPSC-CMs that could be attributed to external causes. One-sided

TIs were calculated for the positive controls, whereas for vehicles two-sided tolerance intervals were applied. Note that there is certain minimal sample size (*n* of experiments) needed for non-parametric TIs based on Wilks' approach to achieve given confidence of 95% (when population coverage is 90%). For one-sided interval, at least 29 samples are required, while two-sided interval needs 46 samples at minimum (Krishnamoorthy et al, 2009).

**Supplemental References**


Ando, H., Yoshinaga, T., Yamamoto, W., Asakura, K., Uda, T., Taniguchi, T., Ojima, A., Shinkyo, R., Kikuchi, K., Osada, T., et al., (2017). A new paradigm for drug-induced torsadogenic risk assessment using human iPS cell-derived cardiomyocytes. J. Pharmacol. Toxicol. Methods. 84, 111-127.

Appel-Dingemanse, S., Horowitz, A., Campestrini, J., Osborne, S., McLeod, J., (2002). The pharmacokinetics of the novel promotile drug, tegaserod, are similar in healthy subjects—male and female, elderly and young. Aliment. Pharmacol. Ther. 15 (7), 937-944.

Banner, W., D. Vernon, D., D. Minton, S., Dean, J., (1991). Non-linear dobutamine pharmacokinetics in a pediatric population. Crit. Care Med. 19 (7), 871-873.

Camm, A.J., (2006). How does pure heart rate lowering impact on cardiac tolerability? Eur. Heart J. Suppl. 8 (suppl_D), D9-D15.

Czock, D., Keller, F., Heringa, M., Rasche, F.M., (2005). Raloxifene pharmacokinetics in males with normal and impaired renal function. Br. J. Clin. Pharmacol. 59 (4), 479-482.

Harmer, A.R., Valentin, J.P., Pollard, C.E., (2011). On the relationship between block of the cardiac Na(+) channel and drug-induced prolongation of the QRS complex. Br. J. Pharmacol. 164 (2), 260-273.

Krishnamoorthy K., Mathew T. (2009). Statistical Tolerance Regions: Theory, Applications, and Computation. John Wiley & Sons, Hoboken, New Jersey.

Olsson, B., Szamosi, J., (2001). Multiple Dose Pharmacokinetics of a New Once Daily Extended Release Tolterodine Formulation Versus Immediate Release Tolterodine. Clin. Pharmacokinet. 40 (3), 227-235.

Redfern, W.S., Carlsson, L., Davis, A.S., Lynch, W.G., MacKenzie, I., Palethorpe, S., Siegl, P.K.S., Strang, I., Sullivan, A.T., Wallis, R., et al., (2003). Relationships between preclinical cardiac electrophysiology, clinical QT interval prolongation and torsade de pointes for a broad range of drugs: evidence for a provisional safety margin in drug development. Cardiovasc. Res. 58 (1), 32-45.

Roth, W., Bauer, E., Heinzel, G., Cornelissen, P.J.G., Van Tol, R.G.L., Jonkman, J.H.G., Zuiderwijk, P.B.M., (1993). Zatebradine: Pharmacokinetics of a novel heart-rate-lowering agent after intravenous infusion and oral administration to healthy subjects. J. Pharm. Sci. 82 (1), 99-106.

Schulz, M., Iwersen-Bergmann, S., Andresen, H., Schmoldt, A., (2012). Therapeutic and toxic blood concentrations of nearly 1,000 drugs and other xenobiotics. Crit. Care 16 (4), R136.

Thuillez, C., Pussard, E., Bellissant, E., Richer, C., Kechrid, R., Giudicelli, J.F., (1991). Arterial vasodilating profile and biological effects of pinacidil in healthy volunteers. Br. J. Clin. Pharmacol. 31 (1), 33-39.

Wilks, S.S., (1941). Determination of Sample Sizes for Setting Tolerance Limits. Ann. Math. Statist. 12 (1), 91-96.

Yao, X., Anderson, D.L., Ross, S.A., Lang, D.G., Desai, B.Z., Cooper, D.C., Wheelan, P., McIntyre, M.S., Bergquist, M.L., MacKenzie, K.I., et al., (2008). Predicting QT prolongation in humans during early drug development using hERG inhibition and an anaesthetized guinea-pig model. Br. J. Pharmacol. 154 (7), 1446-1456.
