# Supplementary material for: Development of a Human iPSC Cardiomyocyte-Based Scoring System for Cardiac Hazard Identification in Early Drug Safety De-risking
Source: Stem Cell Reports. 2018 Dec 11;11(6):1365–77. doi: 10.1016/j.stemcr.2018.11.007 (PMC6294263; doi:10.1016/j.stemcr.2018.11.007)
Supplement: Document S2. Article plus Supplemental Information [file mmc2.pdf]

Stem Cell Reports
Article
OPEN ACCESS

Development of a Human iPSC Cardiomyocyte-Based Scoring System for Cardiac Hazard Identification in Early Drug Safety De-risking

Ivan Kopljar,[1,3,*] Hua Rong Lu,[1,3,*] Karel Van Ammel,[1] Martin Otava,[2] Fetene Tekle,[2] Ard Teisman,[1] and David J. Gallacher[1]
[1]Global Safety Pharmacology, Non-Clinical Safety, Janssen Research & Development, A Division of Janssen Pharmaceutica NV, Turnhoutseweg 30, 2340 Beerse, Belgium
[2]Statistics and Decision Sciences, Quantitative Sciences, Janssen Research & Development, A Division of Janssen Pharmaceutica NV, Turnhoutseweg 30, 2340 Beerse, Belgium
[3]Co-first author
*Correspondence: ikopljar@its.jnj.com (I.K.), hlu@its.jnj.com (H.R.L.)
https://doi.org/10.1016/j.stemcr.2018.11.007

SUMMARY

Human induced pluripotent stem cell-derived cardiomyocytes (hiPSC-CMs) have emerged as a promising cardiac safety platform, demonstrated by numerous validation studies using drugs with known cardiac adverse effects in humans. However, the challenge remains to implement hiPSC-CMs into cardiac de-risking of new chemical entities (NCEs) during preclinical drug development. Here, we used the calcium transient screening assay in hiPSC-CMs to develop a hazard score system for cardiac electrical liabilities. Tolerance interval calculations and evaluation of different classes of cardio-active drugs enabled us to develop a weighted scoring matrix. This approach allowed the translation of various pharmacological effects in hiPSC-CMs into a single hazard label (no, low, high, or very high hazard). Evaluation of 587 internal NCEs and good translation to *ex vivo* and *in vivo* models for a subset of these NCEs highlight the value of the cardiac hazard scoring in facilitating the selection of compounds during early drug safety screening.

INTRODUCTION

Early assessment of cardiac safety liabilities within drug discovery and development is essential to advance promising new chemical entities (NCEs) into clinical evaluation. As such, late-stage attrition due to cardiac safety could be mostly avoided, reducing the potential risk for participants in clinical studies and the costs of getting to this stage. The primary focus of cardiac safety within the current regulatory guidelines is to avoid drug-induced, potentially life-threatening arrhythmias such as torsades de pointes (TdP) (Gintant et al., 2016). TdP is associated with prolonged repolarization of the cardiac action potential, which is observed as prolongation of the QT interval of the electrocardiogram. Inhibition of the hERG channel (gene: *KCNH2*), a voltage-gated K^+ channel that conveys the cardiac rapid delayed rectifier potassium current (I_{Kr}), is the main mechanism associated with drug-induced QT prolongation.

Cardiac action potentials are mediated by multiple ionic currents that can alter the cardiac excitability and function of the heart. In addition to inhibiting hERG, drugs can influence electrophysiological function via various cardiac targets such as cardiac sodium current (I_{Na}), calcium current (I_{Ca}), pacemaker current (I_f), ATP-sensitive potassium currents (I_{KATP}), slow delayed potassium current (I_{Ks}), and calcium-handling proteins, such as Na^+/K^+ ATPases and ryanodine receptors. Beyond QT prolongation, these additional pharmacological actions can result in drug-induced cardiac liabilities such as QT shortening and QRS widening, which are also associated with bradycardia and cardiac arrest, and non-TdP ventricular tachycardia or ventricular fibrillation. These cardiac liabilities not related to prolongation of repolarization also need to be considered during cardiac safety evaluation in pharmaceutical research and development (R&D) (Lu et al., 2008, 2010).

During drug discovery, early potential hazard identification is generally evaluated through binding or functional assays for hERG and other ion channels. However, these assays lack the complexity of an integrated cardiac cellular system (e.g., cardiomyocytes) and provide only indirect insights into potential cardiac electrophysiological actions of compounds. Alternative and/or follow-up studies that do possess the required complex interplay of different ion channels are generally based on animal models. These models have a low throughput, are labor and cost inefficient, and raise concerns about species translation and consistency with the 3R's (reduce, replace, and refine) concept of animal use. Recently, human induced pluripotent stem cell-derived cardiomyocytes (hiPSC-CMs) have emerged as a promising human-derived cardiac *in vitro* platform that can be used in preclinical safety evaluation. The potential of hiPSC-CMs has been recognized and supported by the Comprehensive *In Vitro* Proarrhythmia Assay (CiPA) initiative, with the aim to reshape the existing regulatory guidelines to help identify the torsadogenic (proarrhythmic) risk of drugs (Colatsky et al., 2016). Consequently, hiPSC-CMs have been extensively evaluated mainly for drug-induced QT prolongation and TdP risk. Nevertheless, hiPSC-CMs represent a relevant cardiac

This is an open access article under the CC BY license (http://creativecommons.org/licenses/by/4.0/).

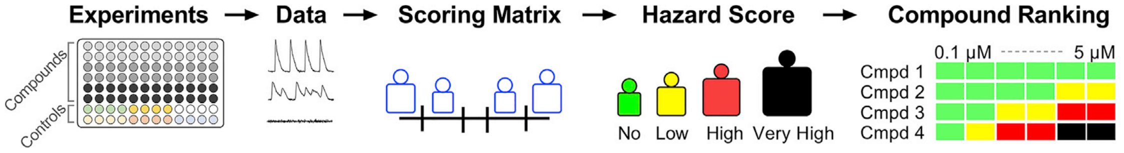

Figure 1. Concept of Cardiac Hazard Identification

Compounds were tested in hiPSC-CMs using a calcium fluorescence imaging assay. Effects on hiPSC-CMs are evaluated through different parameters, which are then fed into a scoring matrix that translates the data into a hazard level. This approach allows a concentration-dependent hazard identification and ranking of drug candidates.

model containing the ionic currents that shape the cardiac action potential. This could allow cardiac hazard identification for different pharmacological mechanisms, beyond hERG or QT prolongation, and positioning of hiPSC-CMs as a versatile early preclinical safety de-risking screening tool. One of the routinely applied measurement methods is calcium transient imaging using calcium-sensitive indicators (Broyles et al., 2018). Intracellular calcium transients reflect the rise and decay of cytosolic Ca^{2+} during a cardiac action potential. Imaging of the internal calcium transients has been shown to reflect action potential duration (APD) and arrhythmic events in hiPSC-CMs (Spencer et al., 2014). Early afterdepolarization (EAD)-like events in hiPSC-CMs, detected with calcium imaging in the form of additional (abnormal) calcium spikes or bursts during a single calcium transient cycle, may represent a potential surrogate for TdP risk in humans. Studies have shown the translational value of the calcium transient-based hiPSC-CM (CTCM) assay by evaluating drugs with known cardiac liabilities in humans (Bedut et al., 2016; Dempsey et al., 2016; Lu et al., 2015; Rast et al., 2015; Watanabe et al., 2017; Zeng et al., 2016). Furthermore, hiPSC-CMs are suggested to show more relevant pharmacological responses in comparison with existent hERG and certain non-human APD or isolated heart assays (Takasuna et al., 2017).

The CTCM assay allows for medium- to high-throughput evaluation of hundreds to thousands of NCEs per year. However, one of the main challenges that remains is assessment of the translational potential of hiPSC-CM assays and implementation for hazard identification and decision-making during (early) preclinical drug discovery. Therefore, a straightforward approach is needed for interpretation of large datasets from hiPSC-CMs. Ideally, the high-throughput CTCM data are converted into an integrated score that can then be used to rank compounds based on hazard level as a function of concentration. The present study illustrates the scientific approaches used to develop and validate a hazard scoring system and conveys opportunities to apply such an approach in other laboratories, and to other technologies and models in early R&D safety assessment.

RESULTS

Fundamentals of Cardiac Hazard Scoring

The goal of our current work was to translate pharmacological effects on hiPSC-CMs using a phenotypic readout (e.g., calcium imaging) into a cardiac hazard score for NCEs being considered for further drug development (Figure 1). Cardiac hazard scoring for the tested compound at a given concentration was differentiated into the following classes (color labels): "no" (green), "low" (yellow), "high" (red), and "very high" (black). No hazard labeling indicates compound effects within the vehicle variability. Low hazard suggests effects outside of the vehicle variability, with likely no or limited risk. High hazard suggests a strong concern that could potentially lead to cardiac adverse effects in the clinic, whereas the very high hazard identifies cardiac arrhythmias, such as torsadogenic risk (e.g., TdP), which could potentially lead to life-threatening events. The respective hazard class was determined by a cumulative scoring of individual parameters using a scoring matrix, which was based on the following three fundamental components: (1) selection of the relevant parameters, (2) defining the cutoffs for level of effect, and (3) defining weighted points per parameter for level of hazard potential.

Characteristics of Calcium Transients in hiPSC-CMs

The CTCM assay is a medium- to high-throughput screening tool used for early cardiac safety testing of compounds. Fluorescence imaging of intracellular calcium dynamics (i.e., calcium transients) enabled the evaluation of the spontaneous beating properties of hiPSC-CMs (Figure 2A) using the following parameters: (1) calcium transient duration at 90% of decay following the peak amplitude (CTD_{90}), (2) beat rate (BR) representing the number of calcium transients (i.e., beats) per minute, and (3) amplitude (Amp), representing the difference between the minimum and the maximum (peak) calcium signal. Figure S1 shows the baseline CTD_{90} and BR values for 23,183 individual experiments used for this study. Although the histograms reflect a Gaussian distribution, the range of CTD_{90} and BR values also showed the expected

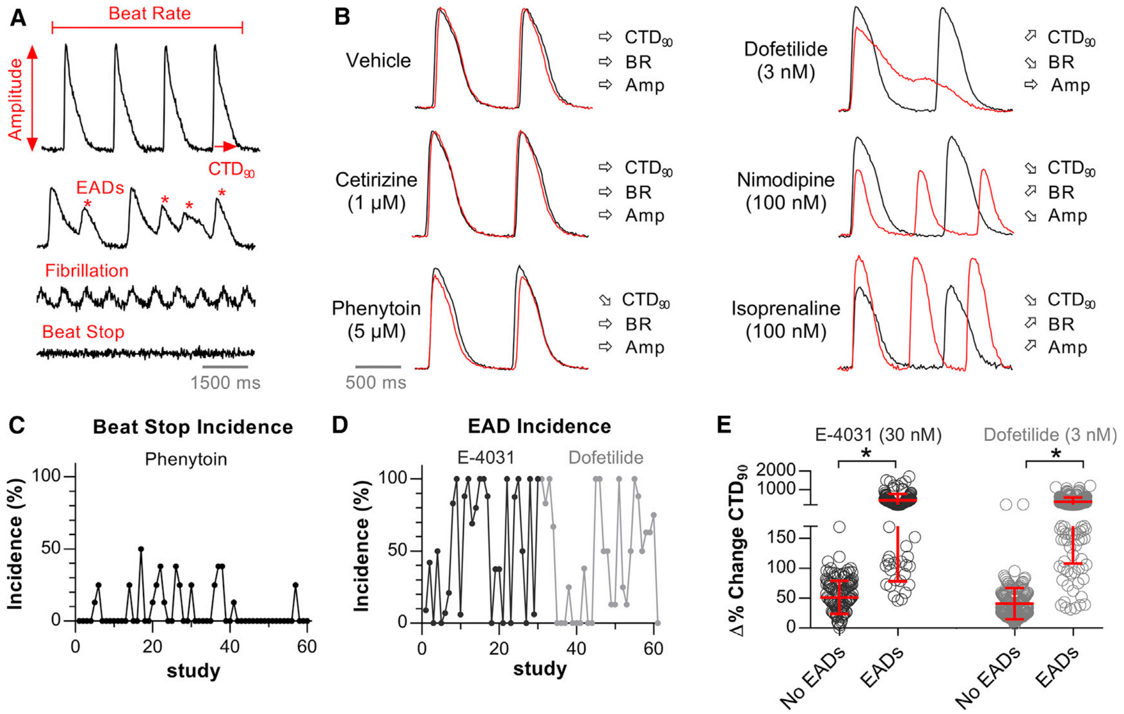

Figure 2. Characteristics and Pharmacological Response of hiPSC-CMs

(A) Representative calcium transient recording allowing evaluation of CTD_{90}, BR, and Amp. Abnormal functioning of hiPSC-CMs was observed in the form of EAD-like events, fibrillation-like events, and beating arrest.

(B) Representative calcium transients showing the 30-min effect (red tracing) of vehicle and control drugs compared with their respective baseline (black tracing). E-4031 effects (not shown) were similar to dofetilide.

(C) Incidence of beat stop (% of wells) after a 30-min incubation with phenytoin at 5 μM. Each study represents n = 4–8 independent experiments.

(D) Incidence of EADs (% of wells) developed during a 30-min incubation with E-4031 (30 nM) or dofetilide (3 nM).

(E) Relationship between the size of CTD_{90} prolongation and EAD occurrence for E-4031 (EAD, n = 150; no EAD, n = 113) and dofetilide (EAD, n = 179; no EAD, n = 193). n represents independent experiments. Error bars represent SD. $^*p < 0.0001$, Mann-Whitney test.

heterogeneity in hiPSC-CMs, reflecting a mixture of different cell types (e.g., ventricular, atrial, and nodal-like) within the syncytium and batch-to-batch and experimental variability. Furthermore, we used uncorrected CTD_{90} values since BR-dependent correction of CTD_{90} was not optimal for our purposes (Figure S2).

For quality control of hiPSC-CMs, each test plate (i.e., 96 unique experiments) contained vehicle treatments together with several control drugs (cetirizine, phenytoin, isoprenaline, nimodipine, and dofetilide or E-4031). These drugs represent different pharmacological classes and were used as a pharmacological reference set for the development of the hazard scoring system. Cetirizine is a true negative control without any clinical cardiac liabilities. Phenytoin is a sodium channel blocker for antiepileptic treatment, with class Ib antiarrhythmic properties, but is not known to be overtly proarrhythmic in the clinic. Nimodipine is a calcium channel antagonist and isoprenaline is a beta-adrenergic agonist. Dofetilide, a hERG blocker associated with QT prolongation and proarrhythmic TdP risk in humans, was also evaluated. E-4031, an experimental hERG blocker, was used as a second agent in this class. Effects on hiPSC-CMs were evaluated after a 30-min incubation period and normalized against the respective baseline recording, yielding a Δ% change in CTD_{90}, BR, and Amp.

Scoring Matrix: Relevant Parameters for Cardiac Hazard Identification

Selection of parameters is one of the crucial aspects of the scoring matrix. The parameters need to reflect relevant changes related to pharmacological effects and preferentially differentiate the severity and direction of effect. CTD_{90}, BR, and Amp (Figure 2A) were selected as primary parameters to quantify changes in calcium transients. CTD_{90} is the main parameter, which reflects both APD prolongation and shortening by compounds, and is used as a surrogate for QT interval changes. BR is secondary with

respect to effects on CTD_{90}. However, it is more relevant for pharmacological classes such as adrenergic agonists, which increase the BR of cardiomyocytes. On the other hand, strongly decreased BR can be related to bradycardic actions or effects on action potential propagation. Amp is a parameter that can give insights regarding effects on internal calcium handling. For example, adrenergic stimulation (e.g., isoprenaline) or Ca^{2+} channel agonists (e.g., BAYK8644) will increase the Amp, whereas Ca^{2+} channel antagonists (e.g., nimodipine) will strongly decrease the Amp. In general, various pharmacological mechanisms evoke a specific (directional) effect on the three parameters (Figure 2B).

In addition to the primary beating parameters, we introduced additional parameters into the scoring matrix in the form of "beat stop," "fibrillation-like" events, and "EADs" (Figure 2A). Beat stop is a multi-purpose parameter since it can reflect various effects on hiPSC-CMs. For example, compounds (at high concentrations) can have strong effects on cardiac electrophysiology that can lead to a beating arrest of the syncytium. Although there would be no information on primary parameter effects, beating arrest indicates a relevant pharmacological action in hiPSC-CMs. However, in certain cases an incidence of beat stop ($\leq$50%) can be observed with relatively cardiac-safe compounds such as phenytoin (Figure 2C), due to weak effects on the sodium current, which can alter the spontaneous beating propagation of hiPSC-CMs. Therefore, we introduced three zones for beat stop incidence (based on percentage of wells with beat stop) to differentiate the level of potential hazard of compounds (Figure 4A). A fibrillation-like phenotype can occur when normal propagation of beating becomes discontinuous, leading to impulse reentry and a fibrillation-like state, as reflected by small, rapid-rate calcium transients. Finally, hiPSC-CMs can develop arrhythmic beating, where additional calcium spikes are observed during a calcium transient or before the following one has initiated (i.e., EAD-like events). This behavior is observed with QT-prolonging drugs, which display proarrhythmic risk and can cause life-threatening TdP in humans. Indeed, E-4031 and dofetilide, positive controls for I_{Kr} (hERG) inhibition, provoked such EAD-like events in hiPSC-CMs, but the incidence varied between plates and studies (Figure 2D). Furthermore, we observed a clear overlap in Δ% CTD_{90} effect ranges between the experimental populations without EADs and those with EADs (Figure 2E). This implies that a cutoff for strong CTD_{90} prolongation can be used to distinguish potential QT prolongation that is not necessarily associated with TdP risk. Therefore, we annotated EADs as a separate parameter instead of predicting EADs using changes in CTD_{90}. Hence, studies showing EAD observations are uniquely categorized as very high (black) hazard.

Scoring Matrix: Defining Cutoffs and Weighted Points

The next part of the scoring matrix was the determination of cutoffs between the different effect zones per parameter (Figure 3). The "no effect" zone represents changes in a parameter that are likely within vehicle variability, whereas "mild" and "strong" zones are differentiated bidirectionally (e.g., CTD_{90} shortening and prolongation). The cutoff values are net changes (ΔΔ% changes versus baseline and vehicle) that are based on the statistical tolerance intervals (TIs) (Δ% changes versus baseline). TIs indicate an interval where, with a certain confidence level, a specified proportion of a sampled population falls. Vehicle treatments showed low variability in Δ% change for CTD_{90}, BR, and Amp with respect to baseline (Figure 3). Such low variability should facilitate the defining of the cutoffs and the development of a robust scoring system. The width of the TIs of vehicles was used to characterize the vehicle effects for each plate and assisted the scientific framework defining the two-sided cutoffs to determine the no effect zone for all parameters. Strong bidirectional cutoffs (Figure 3) were based on the calculation of TIs in numerous studies with control drugs (E-4031, dofetilide, nimodipine, and isoprenaline) and determined by correcting (per parameter) the respective TI with centralized TI windows of vehicles. Cutoffs defining mild changes in CTD_{90} were determined through an iterative optimization process (Figure S3). Evaluation of negative controls, QT-prolonging and QT-shortening drugs, allowed us to determine the TIs for effects on CTD_{90} while minimizing false positive scoring. The cutoff for strong BR decrease was based on the ΔΔ% change in BR by ivabradine (a reference drug) at 0.1 μM, a concentration at which the I_f current is inhibited without additional effects on hERG (half maximal inhibitory concentration [IC_{50}] of approximately 2.4 μM).

The last part of the scoring matrix was the inclusion of a weighted points algorithm to differentiate various levels of cardiac hazard (Figure 4). Defining the weighted points was based on the relevance of a parameter (and direction of effect), together with an expected hazard identification for various pharmacological mechanisms of action. The sum of all weighted parameters resulted in a total hazard score that could be translated into a specific hazard label (Figure 4B). Here, an iterative approach (Supplemental Experimental Procedures and Figure S4) was applied to optimize the weighted points and the overall score ranges associated with the different hazard labels.

Validation of the Hazard Scoring System Using Controls and Reference Drugs

Next, the optimized hazard scoring (labeling) was validated on all studies done with positive controls and reference drugs. It is important to note that the control drugs (an initial smaller set used in Figure 3) and the reference drugs

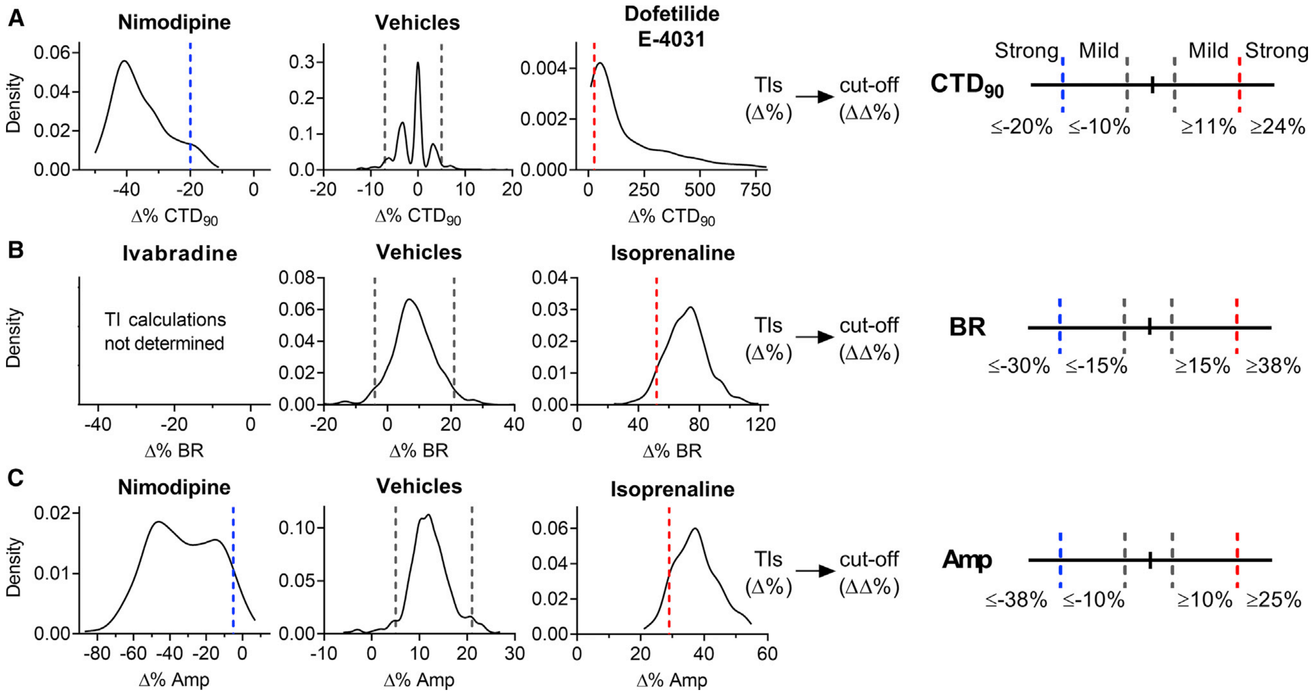

Figure 3. Determination of Cutoffs Using TIs to Develop the Scoring Matrix

Density plots show the TIs (dashed lines within graphs) for vehicles and positive controls to determine bidirectional cutoffs for (A) CTD_{90}, (B) BR, and (C) Amp. TIs on vehicles were applied to aid the process of defining the no effect cutoffs. TIs (Δ%) from control drugs were corrected for vehicle offset to determine the cutoffs (ΔΔ%). Vehicle (n = 639), isoprenaline (0.1 μM; n = 357), nimodipine (0.1 μM; n = 323), E-4031 (30 nM), and dofetilide (3 nM) were combined (n = 387), ivabradine (0.1 μM; n = 6). n indicates the number of independent experiments.

(subset) were used for the development and optimization process of different parts of the scoring system. On the other hand, the validation part represents the overall outcome of the scoring system on all studies done within this analysis. However, it is recommended to use a validation set that is different from the reference (training) set of drugs. Figure 4C shows the hazard distribution for control drugs tested over numerous studies. Cetirizine at 1 μM (16-fold free plasma concentration [free C_{max}]) as a negative control was almost exclusively identified as no hazard (99%), indicating that false positive hazard labeling is very rare. Figure S5 illustrates the influence of varying cutoffs for CTD_{90} on hazard scoring of cetirizine, indicating the importance of cutoff determination using a combination of statistical analysis and experimental evaluation. The sodium blocker phenytoin at 5 μM (free C_{max}) was mainly identified as no hazard (78%) or low hazard (20%), as expected. Nimodipine at 0.1 μM (17-fold free C_{max}) and isoprenaline at 0.1 μM are both cardio-active drugs that were identified within the low (80% and 70%, respectively) and high hazard zones. Dofetilide at 3 nM (2-fold free C_{max}) was correctly identified as very high (80%) or high (14%) hazard. Similar results were observed with E-4031 at 30 nM.

Subsequently, we validated the hazard scoring (as defined in Figure 4) on the 66 reference drugs. These were grouped according to their pharmacological mechanism or level of cardiac risk in the clinic (Figure 5). Concentrations were selected to cover the therapeutic free C_{max}, if applicable. Negative control drugs do not have reports of cardiac liabilities in humans and are expected to be identified as no hazard for concentrations up to 10- to 30-fold the free C_{max}. Indeed, all negative controls were scored as no hazard (green). The only exception was raloxifene (3–10 μM) at clinically non-relevant concentrations, approximately ≥2500-fold the free C_{max} (Figure 6).

Next, we validated the hazard scoring system for drugs that are associated with QT prolongation and a certain degree of TdP risk in the clinic (Figures 5 and 6). Based on the CredibleMeds classification list, these drugs were categorized into three groups: (1) known risk of TdP, (2) possible (theoretical) TdP risk to long QT patients and known to cause QT prolongation, and (3) conditional TdP risk in conditions such as overdose or drug-drug interactions or certain high-risk individuals (Woosley et al., 2018). Most drugs with conditional TdP risk were scored as high hazard at concentrations 10- to 100-fold their free

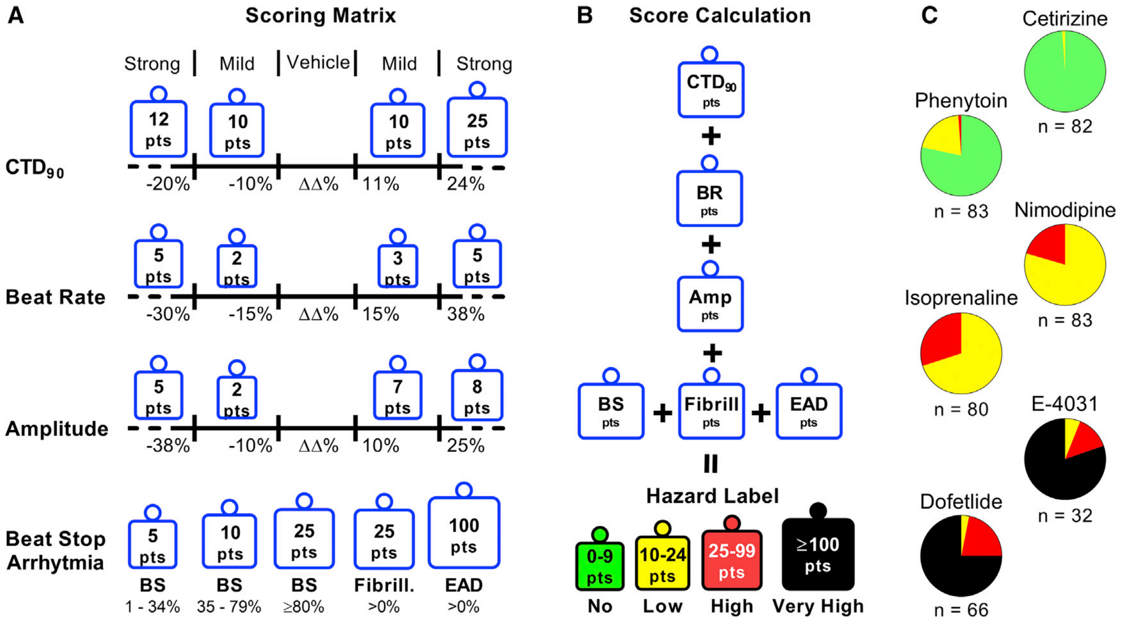

Figure 4. Development of the Scoring Matrix and Calculation Algorithm for Hazard Identification

(A) The scoring matrix represents a points card where for each parameter a weighted score is given dependent on the size and direction of the ΔΔ% effect.

(B) Calculation of hazard level is done through a sum of points across all parameters.

(C) Scoring of controls over multiple studies: cetirizine (1 μM), phenytoin (5 μM), isoprenaline (0.1 μM), nimodipine (0.1 μM), E-4031 (30 nM), dofetilide (3 nM). n indicates the number of studies, each study contained 4–8 independent experiments.

C_{max}. Only olanzapine was not identified with any hazard, most likely since the highest tested concentration (100 nM) is around the free C_{max}. Interestingly, for the conditional TdP drugs, we did not observe any EAD-like arrhythmia (very high hazard). Drugs with possible TdP risk were also identified as high hazard, but with therapeutic ratios similar to those observed with conditional TdP drugs. Sertindole was scored as very high risk at a relatively high concentration (1 μM), approximately 625-fold the free C_{max}. Hence, although conditional and possible TdP drugs were identified as high hazard, arrhythmic events were relatively absent. On the other hand, 77% of known TdP risk drugs were identified as very high hazard. Arrhythmic events were detected between 0.3- and 30-fold the free C_{max} (Figure 6), which clearly indicates a very narrow safety margin for the known TdP drugs. Escitalopram and thioridazine were also identified as high hazard at 3- to 10-fold ratio, whereas citalopram was identified as only low hazard. Other drugs likely associated with a certain degree of QT prolongation (other QT prolongation group) were also identified as low to very high hazard, except for the I_{Ks} blocker JNJ-303.

Although the greatest focus within preclinical safety pharmacology is on QT prolongation, different pharmacological actions on cardiac ionic currents can also result in, e.g., QRS widening or QT shortening. The main effects observed with sodium blockers were a decrease in BR and incidence of beating arrest, which can indeed be explained by I_{Na} block causing decreased BR or complete arrest of action potential propagation in hiPSC-CMs. Such effects are then translated to low or high hazard scores as noted with, for example, phenytoin or mexiletine. Furthermore, most sodium channel blockers also have hERG-inhibiting effects in similar IC_{50} ranges, which then can be readily detected due to CTD_{90} prolongation. Aprindine, propafenone, and propoxyphene show such hazard scoring patterns, whereas encainide, quinidine, and ajmaline caused arrhythmic events. Pinacidil and levcromakalim, two cardiac I_{KATP} channel openers, both caused CTD_{90} shortening in hiPSC-CMs, which allowed identification as potentially hazardous compounds. Nicorandil is a more selective vascular I_{KATP} channel opener and was not shown to have any potential hazard risk in hiPSC-CMs, as expected. The hERG activators ICA-105574 and mallotoxin also caused CTD_{90} shortening, leading to hazard identification.

Calcium channel antagonists evoked strong responses in hiPSC-CMs, showing marked decrease in Amp, together with CTD_{90} shortening and pronounced BR increase. Since the pharmacological activity of this class of compounds is readily identified, optimization of weighted points (through an iterative approach with reference drugs) was

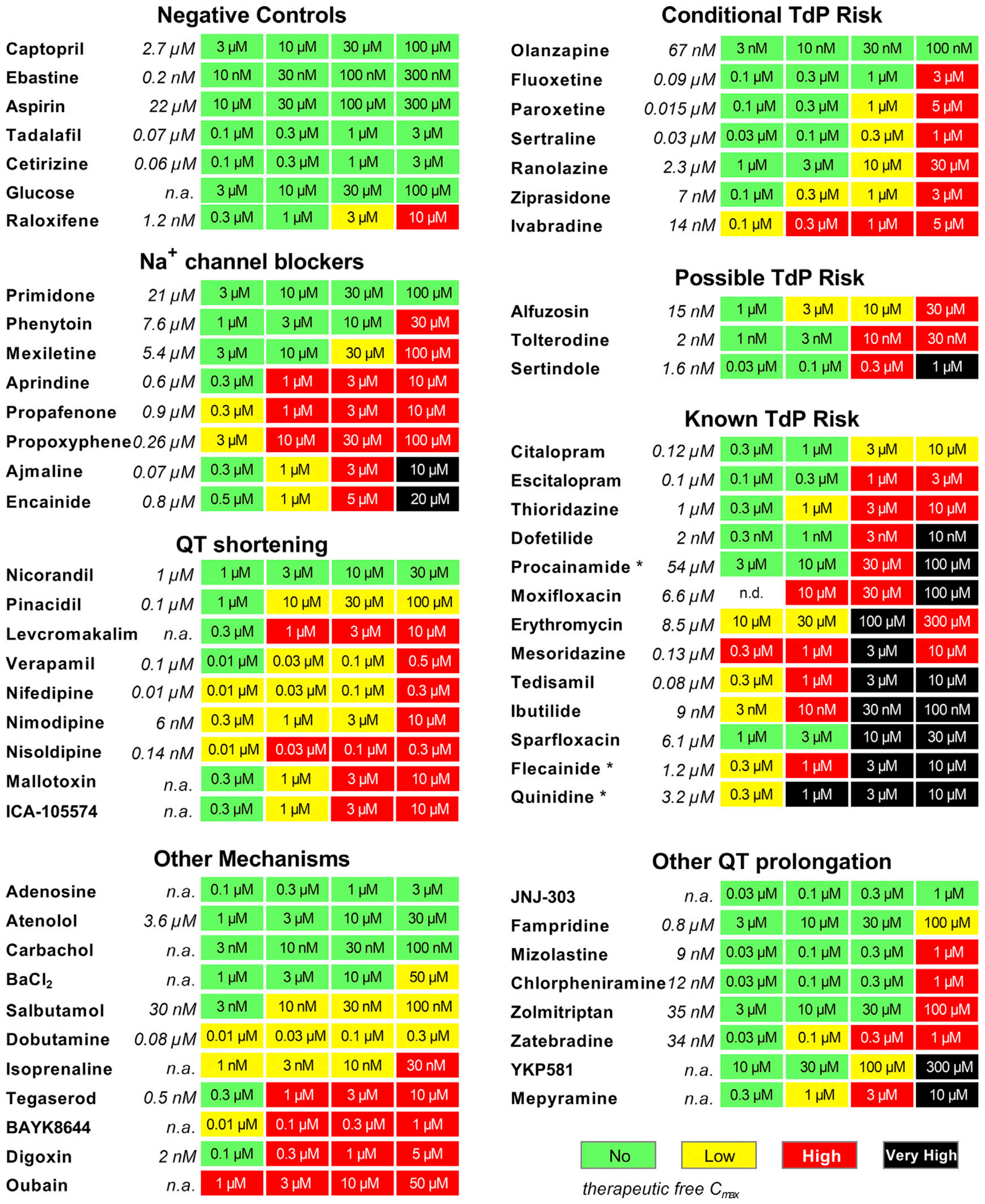

Figure 5. Cardiac Hazard Scoring of Reference Drugs

Conditional TdP, Possible TdP, and Known TdP groups represent drugs that are listed on CredibleMeds. Concentrations were selected based on the therapeutic free C_{max} (shown in italic). *Drugs that can also be categorized as Na^+ channel blockers. n.d., not determined; n.a., not available.

required to minimize over-sensitization of hazard scoring to this activity. Additional mechanisms of pharmacological action were evaluated to broaden the validation exercise. Beta-adrenergic agonists (isoprenaline, dobutamine, and salbutamol) caused strong BR increase and were mainly identified as low or high hazard risk. A high hazard risk was also identified for Na^+/K^+ ATPase inhibitors (digoxin and ouabain), calcium channel activator BAYK8644, and

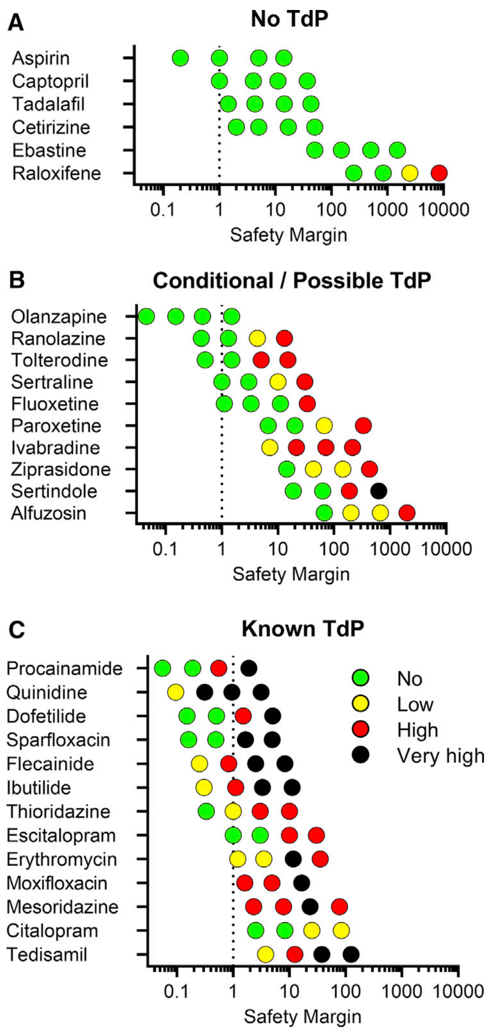

Figure 6. Relationship between Hazard Scoring and the Clinical Safety Margin for TdP Risk

(A–C) (A) No TdP, (B) conditional and possible TdP, and (C) known TdP risk compounds plotted in function of their safety margin (tested concentration/free C_{max}). Dotted line reflects the free C_{max}.

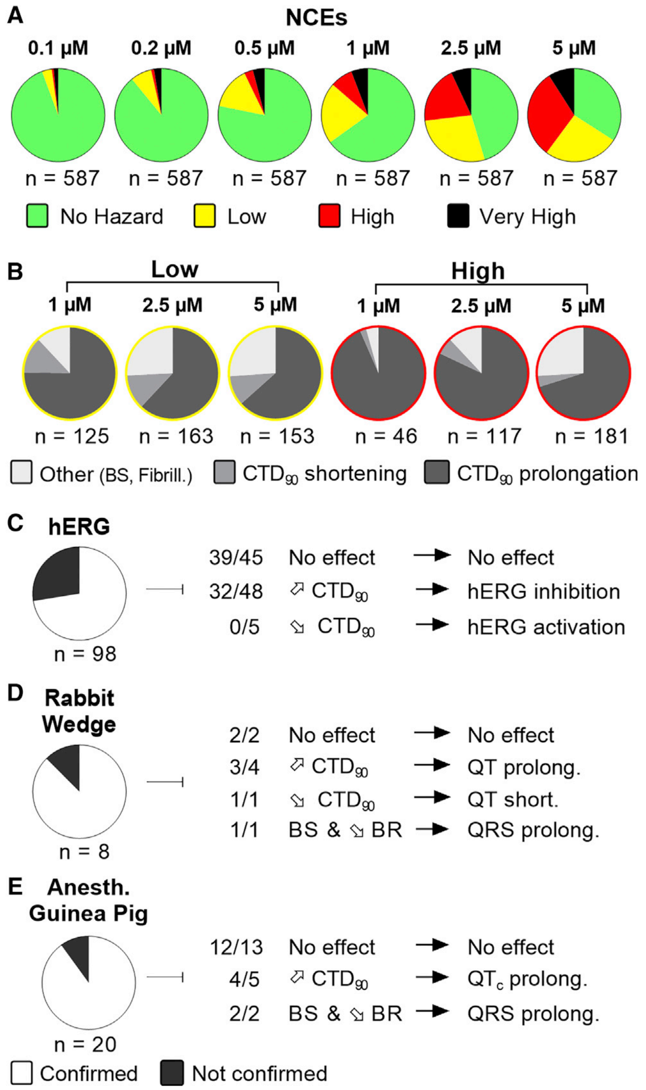

Figure 7. Hazard Evaluation Using the Scoring System on Janssen's NCEs in Discovery

(A) Pie charts showing the concentration-dependent distribution of different hazard levels for NCEs (n = 587).

(B) Analysis of CTD_{90} directional effect for NCEs scored with a low or high hazard at 1, 2.5, and 5 μM.

(C–E) Translational confirmation of the CTCM-derived cardiac hazard scoring of NCEs that were also evaluated in (C) hERG, (D) isolated rabbit wedge, and (E) anesthetized guinea pig models. n indicates the number of NCEs tested in both the CTCM assay and the respective model.

tegaserod, a non-selective 5-HT_4 agonist withdrawn from the market due to adverse cardiovascular events. Adenosine, atenolol, and carbachol were not identified for any potential hazard in the CTCM assay.

Evaluation of the Hazard Potential of NCEs

Following the development and validation of the hazard scoring system, we then evaluated 587 NCEs from current Janssen discovery research programs (Figure 7A). Compounds were tested using a fixed concentration range (0.1–5 μM) that covers the therapeutic free plasma concentrations of most marketed drugs. Hazard evaluation identified that the majority of NCEs were classified within the no hazard group, except for the highest tested concentration of 5 μM. One hundred forty-eight NCEs (25%) were identified as no hazard over the entire tested concentration range. Furthermore, there was a concentration-dependent increase of all hazard levels. At 1 μM and higher, at least 33% of the compounds did show effects associated with a certain hazard level in hiPSC-CMs, with the high hazard

group showing the largest contribution at 5 μM. The very high group, linked to arrhythmic-like EADs, is relatively small but with some incidences also noticed at lower concentrations.

Although the hazard classification is a first overall indicator for cardiac safety evaluation, further in-depth analysis in other models is important to get a better understanding of the respective pharmacological mechanisms leading to an integrated hazard identification. Therefore, we dissected the low and high hazard groups (at 1, 2.5, and 5 μM) with respect to the directional effect on CTD_{90} (Figure 7B). CTD_{90} prolongation seemed to be the largest cause of hazard labeling for all hazard-concentration combinations (Figure 7B). At higher concentrations, hazard identification was based on characteristics other than CTD_{90}. Low hazard labeling was mainly related to beat stop incidence (<80%) and a decrease in BR and Amp (Figure S6A). High hazard labeling was related to 100% beat stop or fibrillation-like events (Figure S6B).

Finally, we evaluated the translational predictability of scored NCEs that have been also tested in our complementary cardiac safety models. Figures 7C–7E show the evaluation of CTCM findings against the hERG assay and the isolated rabbit wedge (*ex vivo*) and anesthetized guinea pig (*in vivo*) models. Changes in relevant CTCM parameters were evaluated against bidirectional percentage change in the I_{Kr} (hERG) current and (corrected) QT and QRS changes in the animal models, using overlapping concentration and/or free C_{max} ranges. The lowest confirmation rate (72%) was found with the hERG model, showing false positive and false negative signals in hERG compared with CTCM. On the other hand, the isolated rabbit wedge (88%) and anesthetized guinea pig (90%) models showed relatively high conformation rates.

DISCUSSION

We have presented the development of a hazard scoring system for preclinical cardiac safety evaluation of NCEs using a hiPSC-CM-based calcium transient screening (CTCM) assay. Ultimately, the application of a visual labeling and ranking of concentration-dependent compound hazard scores allowed us to simplify the interpretation of drug-induced effects on multiple parameters measured from hiPSC-CMs and to apply this approach to select cardiac-safe NCEs in early drug safety de-risking.

Our data show that the CTCM assay can identify drugs targeting various cardiac ion channels and receptors, as reported by previous studies (Bedut et al., 2016; Dempsey et al., 2016; Lu et al., 2015; Rast et al., 2015; Watanabe et al., 2017; Zeng et al., 2016). Statistical analysis of TIs in vehicle and control drug responses enabled the development of a detailed scoring matrix with differentiation of size and direction of effect per parameter. Here, we showed the value of accounting for multiple parameters (CTD_{90}, BR, Amp, beat arrest, fibrillation, and EADs) in spontaneously beating hiPSC-CMs, allowing hazard identification and differentiation of various pharmacological classes of drugs. This is a more comprehensive approach compared with most other studies in hiPSC-CMs, where the focus is set exclusively on APD-related parameters (e.g., CTD_{90}) associated with changes in QT interval. Therefore, our scoring system using the CTCM assay can be applied for cardiac hazard assessment beyond drug-induced QT prolongation, such as QT shortening and conduction slowing.

Nevertheless, drug-induced QT prolongation and proarrhythmic risk (e.g., TdP) remain the largest concerns within cardiovascular safety. We used the CredibleMeds classification of proarrhythmic risk of marketed drugs with evidence of certain degrees of potential TdP risk (Woosley et al., 2018). The hazard identification system could detect torsadogenic (EAD) risk for most of the high-TdP-risk drugs within the 30-fold free C_{max} range. Drugs classified as possible or conditional TdP risk were identified as high hazard or with EADs at much higher free C_{max} ratio. However, studies with dofetilide showed that EAD-like events are not always manifested in hiPSC-CMs, where strong CTD_{90} prolongation sometimes does not cause arrhythmogenic events. We believe that such variability in EAD incidence can be due to the experimental variability and batch-to-batch differences in hiPSC-CMs. However, another more likely reason is the natural complexity of EAD manifestations. Indeed, as an effort to explore the incidence of drug-associated arrhythmias in humans, a clinical pilot study reported an annual incidence of TdP of 4 in 100,000 (Darpö, 2001). This emphasizes the variable incidence of TdP in humans and the challenge to predict TdP in preclinical models. On the other hand, we have not observed any EAD-like event in over 5,000 experiments (wells) treated with negative controls (vehicle and cetirizine), suggesting that EAD-like arrhythmic behavior in hiPSC-CMs can be used as a specific surrogate for TdP. A similar outcome for proarrhythmic prediction in hiPSC-CMs has been shown using a TdP score to assess drugs with varying clinical torsadogenic risk (Ando et al., 2017). Interestingly, drug-induced incidence of EADs in hiPSC-CMs is much higher than the clinical incidence of TdP in humans, which is relatively rare (Tisdale, 2016). Hence, the CTCM assay is more sensitive for TdP risk detection, which is actually preferred for early cardiac safety de-risking in the drug discovery phase. Currently, there is an ongoing effort by pharmaceutical, regulatory, and academic scientists to evaluate the adoption of CiPA, a new preclinical paradigm for assessment of clinical risk for TdP (Colatsky et al., 2016) whereby hiPSC-CM assays are

validated for detection of proarrhythmogenic potential (Millard et al., 2018; Nozaki et al., 2017) together with ion channel and *in silico* approaches. Overall, hiPSC-CMs are a valuable model to evaluate proarrhythmic risk within drug development, but careful considerations are recommended when attempting to differentiate TdP risk from apparently less concerning QT prolongation effect of drugs.

In addition to torsadogenic risk, other cardiac liabilities such as bradycardia, QT shortening, ventricular tachycardia, and fibrillation can also impede the development of a drug (Lu et al., 2008, 2010). These liabilities can be caused by different pharmacological mechanisms. Therefore, the scoring system was developed and evaluated to identify drugs also acting on, e.g., cardiac I_{Na}, I_{Ca}, I_{KATP}, I_f, Na^+/K^+ ATPases, and adrenergic receptors. Combination of a versatile human-based high-throughput approach and a comprehensive hazard identification tool allows an early strategic positioning of the CTCM assay within the cardiovascular safety screening paradigm. This should increase the throughput of compounds compared with the lower throughput and time-consuming *ex vivo* animal action potential models and yield more human-relevant and comprehensive data on cardiac (electrophysiological) liabilities compared with binding assays or single ion channel affinities.

Importantly, the hazard scoring system generates concentration-dependent hazard labeling rather than an overall labeling per compound. The rationale is that the "apparent" clinical (therapeutic) drug concentrations are uncertain during the different phases of preclinical drug development, and therefore it is important to score the NCEs at the level of individual concentrations. Furthermore, our approach also indicates concentration dependency and severity of hazard, facilitating the comparison and decision-making among a larger set of compounds within a specific drug discovery project. The decision-making is also dependent on the therapeutic benefit-risk (e.g., life-threatening versus non-life-threatening less serious diseases), which can influence the interpretation of various hazard labels. Hence, we defined the color label for very high hazard as black, since this indicates potential life-threatening TdP risk due to observed EAD-like events. High hazard (red label) profiles of lead compounds should be assessed in more detail and further investigated with follow-up cardiovascular safety studies. Evaluation of a large sampling of our internal NCEs from across drug discovery projects clearly showed a concentration-dependent increase in different hazards. A more in-depth analysis indicated that both low and high hazard labels were predominantly associated with CTD_{90}-prolonging effects. This might be related to previous findings showing that many NCEs display hERG-inhibiting effects around 5 μM (Lu et al., 2008). Validation of the hazard scoring in CTCM against our other cardiac safety models showed that the translational confirmation of CTCM findings was relatively high in the isolated rabbit wedge and anesthetized guinea pig models, indicating good predictivity of the CTCM-based hazard scoring system. Since hiPSC-CMs represent a more complex cardiac model than only I_{Kr}, the translation to the hERG assay was, as expected, lower compared with *in/ex vivo* models.

The scoring system can be applied not only to various calcium-imaging-based assays but also to different detection technologies and experimental models. Using the same principal (i.e., design of the scoring matrix and the hazard labeling), the scoring system could be developed for technologies based on multi-electrode arrays (Yamazaki et al., 2018), voltage-sensitive dyes (Blinova et al., 2017; Lu et al., 2017), impedance (Zhang et al., 2016), and video motion imaging (Kopljar et al., 2017) to comprehensively account (indirectly) for APD-like parameters as well as other pharmacological responses such as BR, beat stop, and EAD-like events. Similarly, different cardiomyocyte models (primary or stem cell-derived) can be used. Furthermore, studies have shown the potential of machine learning approaches in cardiac pharmacology classification (Heylman et al., 2015; Lee et al., 2017). Such approaches might refine the scoring matrix to further optimize the hazard scoring system.

Although the hazard scoring system is a promising tool, we want to emphasize the limitations of our approach. Drug responses possess a certain degree of variability. In our model, this means that variability in (Δ)Δ% effects together with the use of static cutoffs can eventually lead to different hazard labeling (between no/low and low/high). Limitations of hiPSC-CMs and applied technologies should also be taken into consideration. hiPSC-CMs represent a fetal-like phenotype, with a more immature morphology, force contractility, calcium handling, and energy metabolism compared with adult native cardiomyocytes (Yang et al., 2014). With respect to cardiac electrophysiology, calcium channel antagonists evoke strong responses on the BR of hiPSC-CMs, which needs to be considered within the hazard system. On the other hand, I_{Ks} blockers (e.g., JNJ-303) cannot be easily detected in *ex vivo* primary myocytes (Varró et al., 2000) or in hiPSC-CMs, most likely due to the role of I_{Ks} as a repolarization reserve (Braam et al., 2013) and the lack of adrenergic activation *in vitro*. Sodium channel blockers can be detected but not easily differentiated from other pharmacological mechanisms. Calcium transient imaging is an invasive approach in which the calcium dyes can affect the physiology of the model (Bootman et al., 2018), although we have optimized the CTCM assay to minimalize such impacts (Kopljar et al., 2018). In addition, we recommend

timely re-analysis of TIs and experimental variability to account for possible changes in hiPSC-CM cultures or experimental procedures over time. Furthermore, statistical analysis (TIs) should be used only as a supportive tool and not an arbitrary approach for the determination of cut-offs. Finally, we have not examined the application of the hazard scoring on other cell lines. Different hiPSC-CM cell lines can possess experimental and phenotypic variability (Sala et al., 2017), although a cross-site validation study showed that drug categorization for TdP risk was comparable between two commercial cell lines (Blinova et al., 2018).

In summary, the development of a cardiac scoring system using calcium imaging in hiPSC-CMs allows early preclinical hazard identification for NCEs in a high-throughput modality. The system allows a more comprehensive identification of multiple pharmacological actions affecting cardiac electrophysiology in hiPSC-CMs using a composite score and can identify different levels of TdP risk. Furthermore, the methodological approach for devising the hazard scoring system could be applied to other cardiac-related assays as well as other biomedical screening assays for risk quantification.

EXPERIMENTAL PROCEDURES

The *in vivo*/*ex vivo* data analysis in this study originates from animal care and experimental procedures conducted at Janssen Pharmaceutica facilities in accordance with the European Directive of September 22, 2010, regarding the protection of animals that are used for scientific purposes and the Belgian Act of May 29, 2013 on the implication of this directive.

Cell Culture and Reagents

hiPSC-CMs were purchased from Ncardia (Cor.4U cardiomyocytes, Ax-C-HC02-96) as living pre-plated cells seeded onto fibronectin-coated 96-well μClear plates (Greiner Bio-One, No. 655090) at a density (~25,000 cells/well) suited to forming a confluent synchronously beating monolayer. Cor.4U cardiomyocytes represent a mix of 60% ventricular, 30% atrial, and 10% nodal cells according to the cell provider. Cells were cultured with Cor.4U culture medium (Ax-M-HC250) in a humidified incubator at 37°C and 5% CO_2, with medium being changed once a day. On the day of the experiment, the culture medium was replaced with Tyrode's solution (Sigma, No. T2397) supplemented with 10 mM HEPES together with KCl to represent isokalemic (4.2 mM K^+) conditions.

Table S1 contains the purchase information and free C_{max} references for all the compounds used within this work. The preparation of drug solutions is explained in Supplemental Experimental Procedures. The calcium-sensitive fluorescence dye Cal-520 AM (Cat. No. 36,338; AAT Bioquest) was used to capture the intracellular calcium transients in hiPSC-CMs. The protocol was used as described in Kopljar et al. (2018). Briefly, Cal-520 was incubated for 45 min followed by a washout and a 30-min recovery before starting the experiments.

Calcium Transient Measurements

The spontaneous beating activity of hiPSC-CMs was assessed through measurement of the Ca^{2+} fluorescence signal integrated over the whole well. Fluorescence signals were measured using the FDSS/μCell platform and the records subsequently analyzed offline using NOTOCORD-hem software (version 4.3), containing EXT modules and an algorithm developed by XiTechniX to detect beat-by-beat Amp, BR, and CTD_{90} parameters (Figure S7). All wells within a plate were measured simultaneously using the following FDSS/μCell settings: sampling frequency 66.7 Hz, exposure time 14.6 ms, excitation wavelength 480 nm, emission wavelength 540 nm, temperature controlled at 37°C.

First, the experimental plates were put into the FDSS/μCell to stabilize for 10 min. Next, a baseline recording was run for 3 min followed by compound addition. The effect of a compound was recorded (5-min recording time) around 15 and 30 min after compound addition. CTD_{90}, BR, and Amp were quantified for baseline and 30-min compound effects as the median value of all beats (calcium transients) measured within a 1-min interval of the recording. The recording around 15 min was used only for observation of EADs or fibrillation-like events. EADs were manually monitored and evaluated. Beat stop was defined after 30 min in case BR was <5 beats/min. Wells that temporarily stopped beating during compound addition but recovered at the 30-min time point were not defined as beat stop.

Quality Control

Wells showing no beating or non-synchronous beating at baseline were excluded from analysis. Wells with a BR at baseline lower than 30 or higher than 90 beats/min were also excluded. Plates with more than 10% of the wells outside of the BR criteria were entirely excluded. Further quality control of the plates was evaluated using positive control drugs. Plates were excluded under the following criteria: (1) <15 ΔΔ% change on CTD_{90} with dofetilide at 3 nM, (2) <30 ΔΔ% change on BR with isoprenaline at 0.1 μM, and (3) >−10 ΔΔ% change on CTD_{90} with nimodipine at 0.1 μM. In total, 13/256 (5.1%) plates were excluded, 8 plates because of BR criteria and 5 plates because of positive control criteria.

Statistical Analysis

The responses were measured at baseline and at 30 min. Consequently, the Δ% effect was calculated for each experiment and each response using the following formula (example of CTD_{90}):

$$CTD_{90}\Delta\% = 100 \cdot \frac{CTD_{90}(30\ mins) - CTD_{90}(baseline)}{CTD_{90}(baseline)}.$$

Hence, Δ% effect reflects changes at 30 min with respect to the baseline values. To avoid possible influence of plate effect, further adjustment of the data was performed by calculation of ΔΔ% (net) effect, which adjusts the drug effects based on effects observed for vehicle in a given plate. The drug and vehicle effect is represented as the median of the individual experiments observed within the respective plate (n = 4–8 per plate). Hence:

$$CTD_{90}\Delta\Delta\% = CTD_{90}\Delta\%\ (drug) - CTD_{90}\Delta\%\ (vehicle).$$

Non-parametric TIs were calculated with Wilks' approach at 95% confidence level covering 90% of a population (more details are provided in Supplemental Information).

SUPPLEMENTAL INFORMATION

Supplemental Information includes Supplemental Experimental Procedures, seven figures, and one table and can be found with this article online at https://doi.org/10.1016/j.stemcr.2018.11.007.

AUTHOR CONTRIBUTIONS

I.K., H.L., A.T., and D.J.G. conceived the study. I.K. performed and analyzed the calcium transient assay experiments. M.O. and F.T. designed and conducted statistical analysis. I.K. and H.L. developed the hazard scoring system, with input from K.V.A., A.T., and D.J.G. K.V.A. performed the hazard scoring analysis. I.K. wrote the paper. All authors reviewed the manuscript and approved the final version.

ACKNOWLEDGMENTS

The authors wish to thank D. Geyskens and E. Vlaminckx for their technical assistance. The authors also wish to thank all the colleagues from the Global Safety Pharmacology group for their helpful discussions and B. Damiano for the scientific review of the manuscript. The research leading to these results has received support from the Innovative Medicines Initiative Joint Undertaking under grant agreement no. 115439 (StemBANCC), resources of which are composed of a financial contribution from the European Union's Seventh Framework Programme (FP7/2007-2013) and EFPIA companies' in-kind contribution.

Received: June 29, 2018
Revised: November 9, 2018
Accepted: November 9, 2018
Published: December 11, 2018

REFERENCES

Ando, H., Yoshinaga, T., Yamamoto, W., Asakura, K., Uda, T., Taniguchi, T., Ojima, A., Shinkyo, R., Kikuchi, K., Osada, T., et al. (2017). A new paradigm for drug-induced torsadogenic risk assessment using human iPS cell-derived cardiomyocytes. J. Pharmacol. Toxicol. Methods *84*, 111–127.

Bedut, S., Seminatore-Nole, C., Lamamy, V., Caignard, S., Boutin, J.A., Nosjean, O., Stephan, J.P., and Coge, F. (2016). High-throughput drug profiling with voltage- and calcium-sensitive fluorescent probes in human iPSC-derived cardiomyocytes. Am. J. Physiol. Heart Circ. Physiol. *311*, H44–H53.

Blinova, K., Dang, Q., Millard, D., Smith, G., Pierson, J., Guo, L., Brock, M., Lu, H.R., Kraushaar, U., Zeng, H., et al. (2018). International multisite study of human-induced pluripotent stem cell-derived cardiomyocytes for drug proarrhythmic potential assessment. Cell Rep. *24*, 3582–3592.

Blinova, K., Stohlman, J., Vicente, J., Chan, D., Johannesen, L., Hortigon-Vinagre, M.P., Zamora, V., Smith, G., Crumb, W.J., Pang, L., et al. (2017). Comprehensive translational assessment of human-induced pluripotent stem cell derived cardiomyocytes for evaluating drug-induced arrhythmias. Toxicol. Sci. *155*, 234–247.

Bootman, M.D., Allman, S., Rietdorf, K., and Bultynck, G. (2018). Deleterious effects of calcium indicators within cells; an inconvenient truth. Cell Calcium *73*, 82–87.

Braam, S.R., Tertoolen, L., Casini, S., Matsa, E., Lu, H.R., Teisman, A., Passier, R., Denning, C., Gallacher, D.J., Towart, R., et al. (2013). Repolarization reserve determines drug responses in human pluripotent stem cell derived cardiomyocytes. Stem Cell Res. *10*, 48–56.

Broyles, C., Robinson, P., and Daniels, M. (2018). Fluorescent, bioluminescent, and optogenetic approaches to study excitable physiology in the single cardiomyocyte. Cells *7*, 51.

Colatsky, T., Fermini, B., Gintant, G., Pierson, J.B., Sager, P., Sekino, Y., Strauss, D.G., and Stockbridge, N. (2016). The comprehensive in vitro proarrhythmia assay (CiPA) initiative—update on progress. J. Pharmacol. Toxicol. Methods *81*, 15–20.

Darpö, B. (2001). Spectrum of drugs prolonging QT interval and the incidence of torsades de pointes. Eur. Heart J. Suppl. *3*(Suppl. K), K70–K80.

Dempsey, G.T., Chaudhary, K.W., Atwater, N., Nguyen, C., Brown, B.S., McNeish, J.D., Cohen, A.E., and Kralj, J.M. (2016). Cardiotoxicity screening with simultaneous optogenetic pacing, voltage imaging and calcium imaging. J. Pharmacol. Toxicol. Methods *81*, 240–250.

Gintant, G., Sager, P.T., and Stockbridge, N. (2016). Evolution of strategies to improve preclinical cardiac safety testing. Nat. Rev. Drug Discov. *15*, 457–471.

Heylman, C., Datta, R., Sobrino, A., George, S., and Gratton, E. (2015). Supervised machine learning for classification of the electrophysiological effects of chronotropic drugs on human induced pluripotent stem cell-derived cardiomyocytes. PLoS One *10*, e0144572.

Kopljar, I., De Bondt, A., Vinken, P., Teisman, A., Damiano, B., Goeminne, N., Van den Wyngaert, I., Gallacher, D.J., and Lu, H.R. (2017). Chronic drug-induced effects on contractile motion properties and cardiac biomarkers in human induced pluripotent stem cell-derived cardiomyocytes. Br. J. Pharmacol. *174*, 3766–3779.

Kopljar, I., Hermans, A.N., Teisman, A., Gallacher, D.J., and Lu, H.R. (2018). Impact of calcium-sensitive dyes on the beating properties and pharmacological responses of human iPS-derived cardiomyocytes using the calcium transient assay. J. Pharmacol. Toxicol. Methods *91*, 80–86.

Lee, E.K., Tran, D.D., Keung, W., Chan, P., Wong, G., Chan, C.W., Costa, K.D., Li, R.A., and Khine, M. (2017). Machine learning of human pluripotent stem cell-derived engineered cardiac tissue contractility for automated drug classification. Stem Cell Reports *9*, 1560–1572.

Lu, H.R., Hortigon-Vinagre, M.P., Zamora, V., Kopljar, I., De Bondt, A., Gallacher, D.J., and Smith, G. (2017). Application of optical action potentials in human induced pluripotent stem cells-derived cardiomyocytes to predict drug-induced cardiac arrhythmias. J. Pharmacol. Toxicol. Methods *87*, 53–67.

Lu, H.R., Rohrbacher, J., Vlaminckx, E., Ammel, K.V., Yan, G.X., and Gallacher, D.J. (2010). Predicting drug-induced slowing of

conduction and pro-arrhythmia: identifying the 'bad' sodium current blockers. Br. J. Pharmacol. *160*, 60–76.

Lu, H.R., Vlaminckx, E., Hermans, A.N., Rohrbacher, J., Van Ammel, K., Towart, R., Pugsley, M., and Gallacher, D.J. (2008). Predicting drug-induced changes in QT interval and arrhythmias: QT-shortening drugs point to gaps in the ICHS7B guidelines. Br. J. Pharmacol. *154*, 1427–1438.

Lu, H.R., Whittaker, R., Price, J.H., Vega, R., Pfeiffer, E.R., Cerignoli, F., Towart, R., and Gallacher, D.J. (2015). High throughput measurement of Ca++ dynamics in human stem cell-derived cardiomyocytes by kinetic image cytometery: a cardiac risk assessment characterization using a large panel of cardioactive and inactive compounds. Toxicol. Sci. *148*, 503–516.

Millard, D., Dang, Q., Shi, H., Zhang, X., Strock, C., Kraushaar, U., Zeng, H., Levesque, P., Lu, H.-R., Guillon, J.-M., et al. (2018). Cross-site reliability of human induced pluripotent stem-cell derived cardiomyocyte based safety assays using microelectrode arrays: results from a blinded CiPA pilot study. Toxicol. Sci. *164*, 550–562.

Nozaki, Y., Honda, Y., Watanabe, H., Saiki, S., Koyabu, K., Itoh, T., Nagasawa, C., Nakamori, C., Nakayama, C., Iwasaki, H., et al. (2017). CSAHi study-2: validation of multi-electrode array systems (MEA60/2100) for prediction of drug-induced proarrhythmia using human iPS cell-derived cardiomyocytes: assessment of reference compounds and comparison with non-clinical studies and clinical information. Regul. Toxicol. Pharmacol. *88*, 238–251.

Rast, G., Weber, J., Disch, C., Schuck, E., Ittrich, C., and Guth, B.D. (2015). An integrated platform for simultaneous multi-well field potential recording and Fura-2-based calcium transient ratiometry in human induced pluripotent stem cell (hiPSC)-derived cardiomyocytes. J. Pharmacol. Toxicol. Methods *75*, 91–100.

Sala, L., Bellin, M., and Mummery, C.L. (2017). Integrating cardiomyocytes from human pluripotent stem cells in safety pharmacology: has the time come? Br. J. Pharmacol. *174*, 3749–3765.

Spencer, C.I., Baba, S., Nakamura, K., Hua, Ethan A., Sears, Marie A.F., Fu, C.-c., Zhang, J., Balijepalli, S., Tomoda, K., Hayashi, Y., et al. (2014). Calcium transients closely reflect prolonged action potentials in iPSC models of inherited cardiac arrhythmia. Stem Cell Reports *3*, 269–281.

Takasuna, K., Asakura, K., Araki, S., Ando, H., Kazusa, K., Kitaguchi, T., Kunimatsu, T., Suzuki, S., and Miyamoto, N. (2017). Comprehensive in vitro cardiac safety assessment using human stem cell technology: overview of CSAHi HEART initiative. J. Pharmacol. Toxicol. Methods *83*, 42–54.

Tisdale, J.E. (2016). Drug-induced QT interval prolongation and torsades de pointes: role of the pharmacist in risk assessment, prevention and management. Can. Pharm. J. (Ott) *149*, 139–152.

Varró, A., Baláti, B., Iost, N., Takács, J., Virág, L., Lathrop, D.A., Csaba, L., Tálosi, L., and Papp, J.G. (2000). The role of the delayed rectifier component IKs in dog ventricular muscle and Purkinje fibre repolarization. J. Physiol. *523*, 67–81.

Watanabe, H., Honda, Y., Deguchi, J., Yamada, T., and Bando, K. (2017). Usefulness of cardiotoxicity assessment using calcium transient in human induced pluripotent stem cell-derived cardiomyocytes. J. Toxicol. Sci. *42*, 519–527.

Woosley, R.L., Black, K., Heise, C.W., and Romero, K. (2018). CredibleMeds.org: what does it offer? Trends Cardiovasc. Med. *28*, 94–99.

Yamazaki, D., Kitaguchi, T., Ishimura, M., Taniguchi, T., Yamanishi, A., Saji, D., Takahashi, E., Oguchi, M., Moriyama, Y., Maeda, S., et al. (2018). Proarrhythmia risk prediction using human induced pluripotent stem cell-derived cardiomyocytes. J. Pharmacol. Sci. *136*, 249–256.

Yang, X., Pabon, L., and Murry, C.E. (2014). Engineering adolescence: maturation of human pluripotent stem cell-derived cardiomyocytes. Circ. Res. *114*, 511–523.

Zeng, H., Roman, M.I., Lis, E., Lagrutta, A., and Sannajust, F. (2016). Use of FDSS/μCell imaging platform for preclinical cardiac electrophysiology safety screening of compounds in human induced pluripotent stem cell-derived cardiomyocytes. J. Pharmacol. Toxicol. Methods *81*, 217–222.

Zhang, X., Guo, L., Zeng, H., White, S.L., Furniss, M., Balasubramanian, B., Lis, E., Lagrutta, A., Sannajust, F., Zhao, L.L., et al. (2016). Multi-parametric assessment of cardiomyocyte excitation-contraction coupling using impedance and field potential recording: a tool for cardiac safety assessment. J. Pharmacol. Toxicol. Methods *81*, 201–216.

Stem Cell Reports, Volume *11*

Supplemental Information

Development of a Human iPSC Cardiomyocyte-Based Scoring System for Cardiac Hazard Identification in Early Drug Safety De-risking

Ivan Kopljar, Hua Rong Lu, Karel Van Ammel, Martin Otava, Fetene Tekle, Ard Teisman, and David J. Gallacher

Supplemental Figures

Figure S1

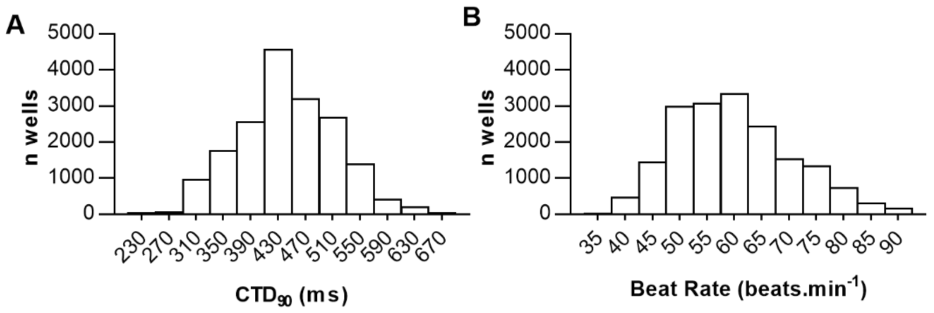

Figure S1. **Beating properties of hiPSC-CMs. Related to Figure 2.** Histograms reflecting a Gaussian distribution of baseline beating properties (CTD_{90} and BR). Data is based on n = 23,183 independent experiments used within the entire analysis that have passed the quality control criteria.

Figure S2

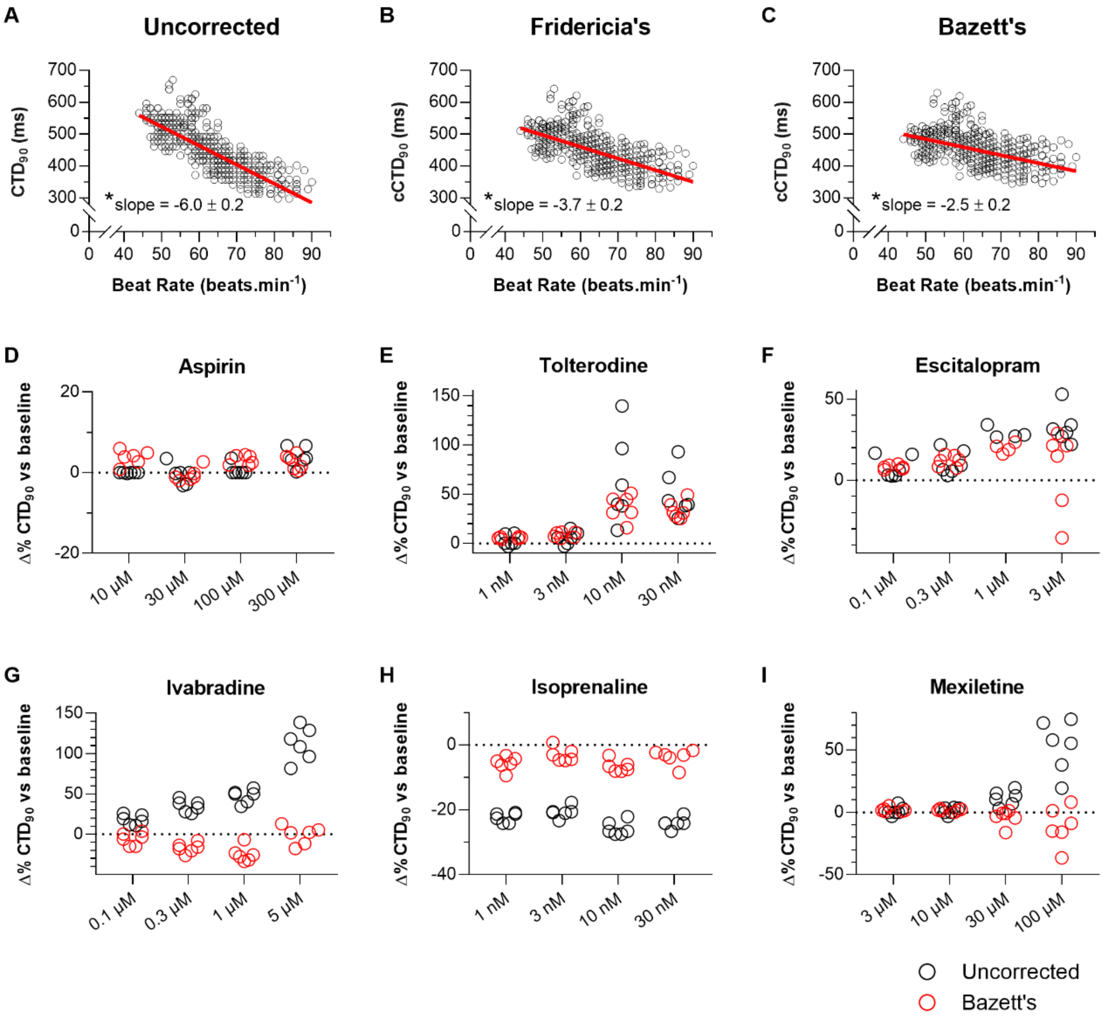

Figure S2. **Correction of CTD_{90} in spontaneously beating hiPS-CMs. Related to Figure 2.** A) Relationship between CTD_{90} and BR using uncorrected baseline experiments, fitted with a linear regression (red line). Correction of CTD_{90} values from A) using B) Fridericia's and C) Bazett's correction formulas still displays a correlation between corrected (c)CTD_{90} and BR. n = 728. Slope is represented as best-fit value ± S.E.M. *: significant deviation from zero; p<0001. D-I) Examples of drug-related Δ% changes for Bazett-corrected compared to uncorrected CTD_{90} values. n = 6 independent experiments. Note for certain drugs strong (directional) differences between uncorrected and corrected data points.

Figure S3

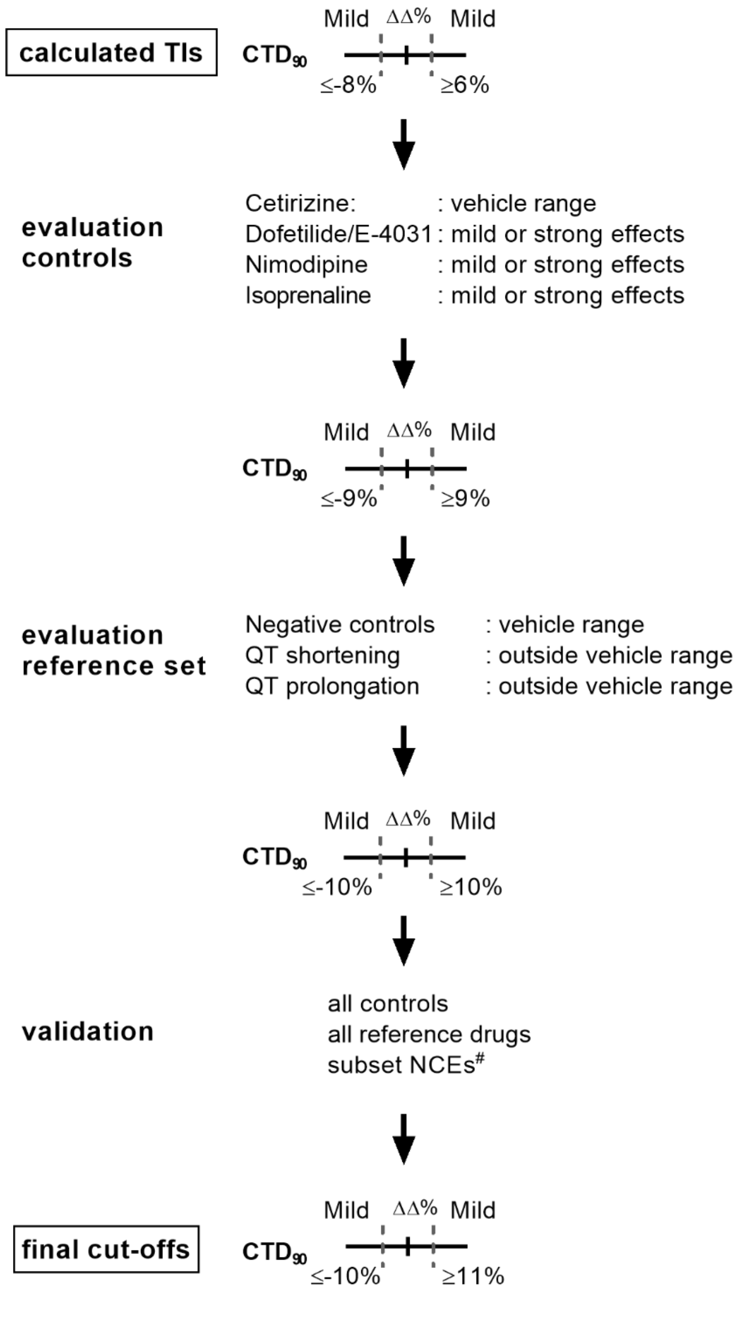

Figure S3. **Optimization of mild CTD_{90} cut-offs. Related to Figure 4.** Process of optimization of mild changes in CTD_{90} starting from calculated TIs. #A subset of NCEs was used to evaluate possible false positive hazard labeling in NCEs. Based on the validation step, the cut-off for CTD_{90} prolongation was set to 11%. This was considered the cut-off where false positives are minimalized whereas the sensitivity for true positive signals is still sufficient.

Figure S4

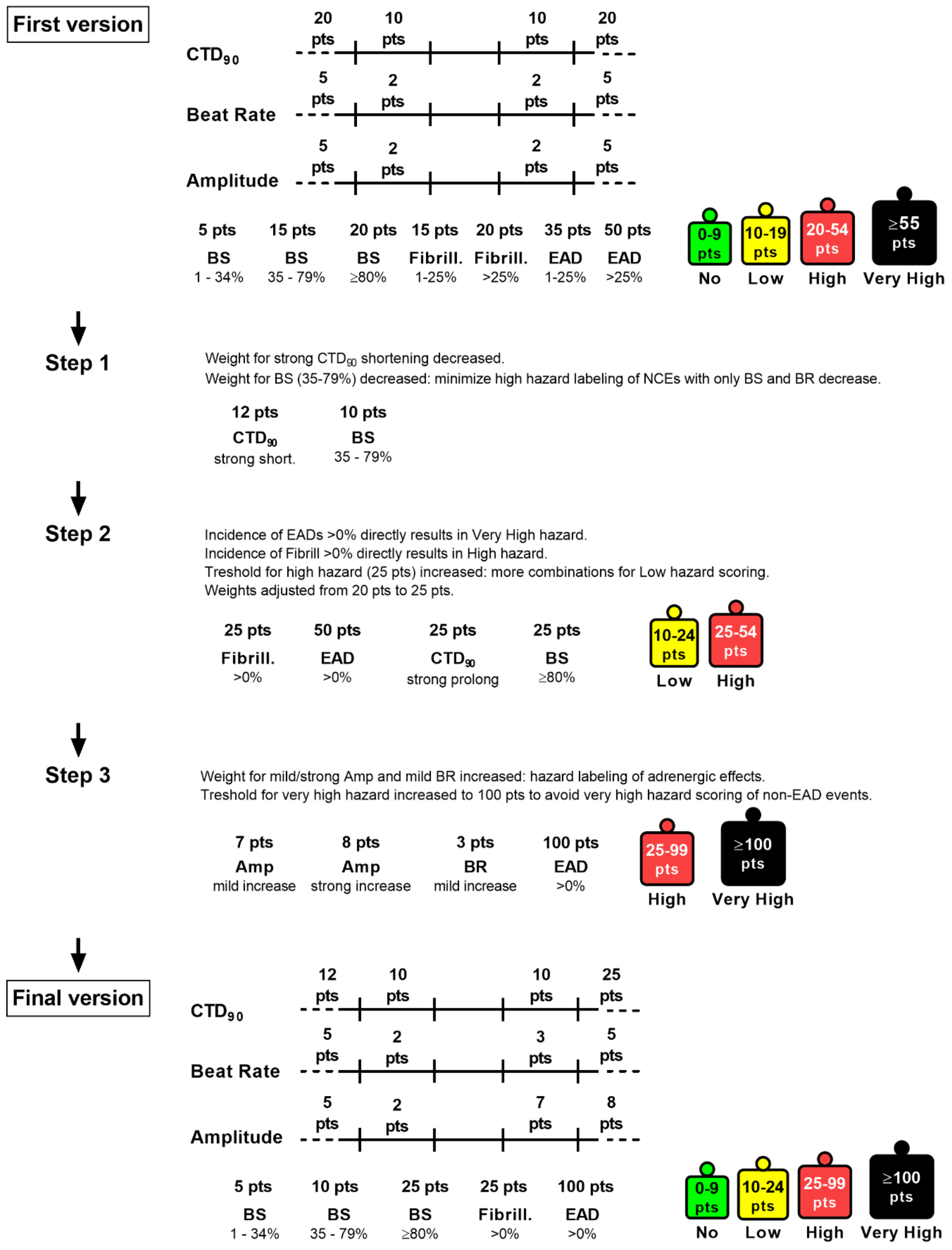

Figure S4. **Optimization of the scoring matrix. Related to Figure 4.** Process of optimization of the weights and hazard labels.

Figure S5

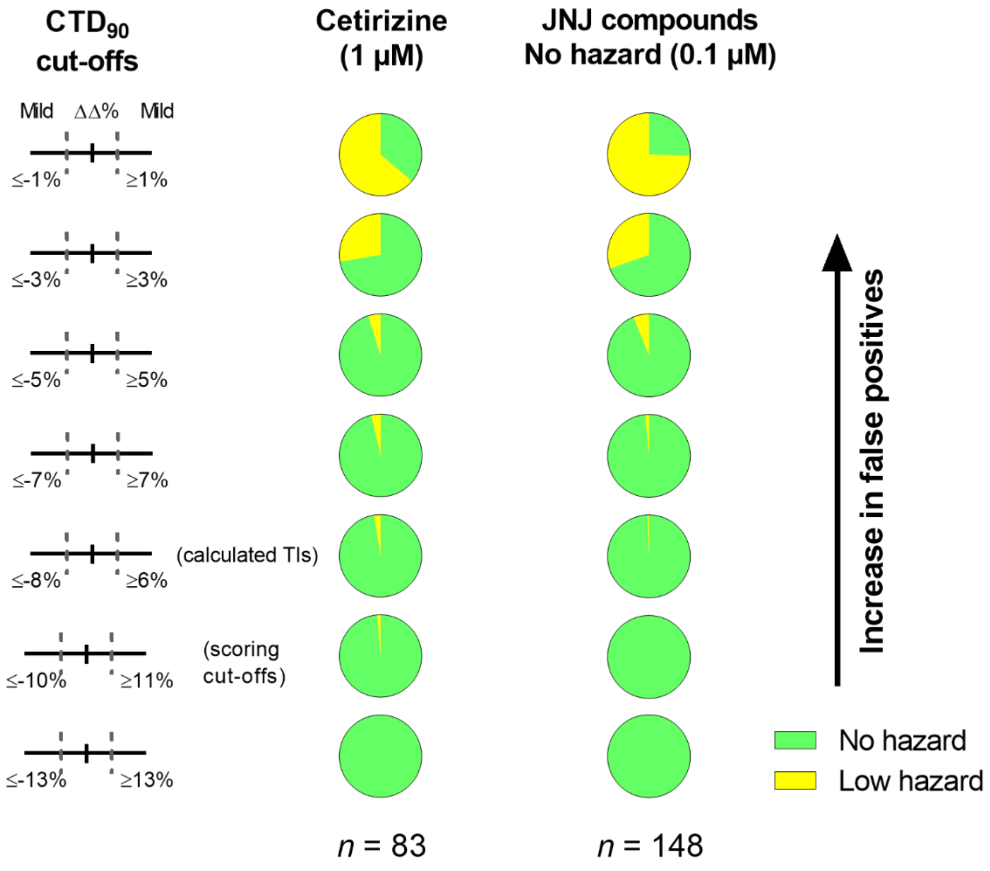

Figure S5. The effect of CTD_{90} cut-off selection on false positive hazard labeling. Related to Figure 4. Different cut-offs shown as ΔΔ% changes were evaluated on the negative control cetirizine (1 µM) and on Janssen's NCEs (0.1 µM) which showed an overall no hazard profile within the tested concentration range (0.1–5 µM).

Figure S6

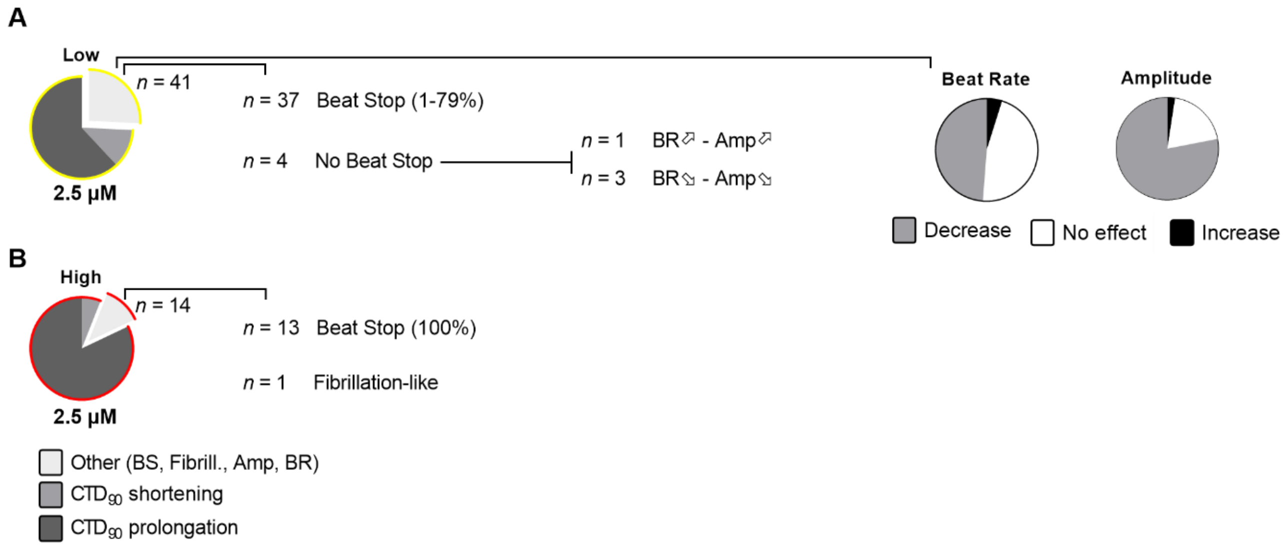

Figure S6. **The impact of non-CTD$_{90}$ parameters on hazard labeling of NCEs. Related to Figure 7.** Detailed analysis of A) low and B) high hazard labeling of NCEs (2.5µM) not related to CTD$_{90}$ changes. n indicates the number of evaluated NCEs.

Figure S7

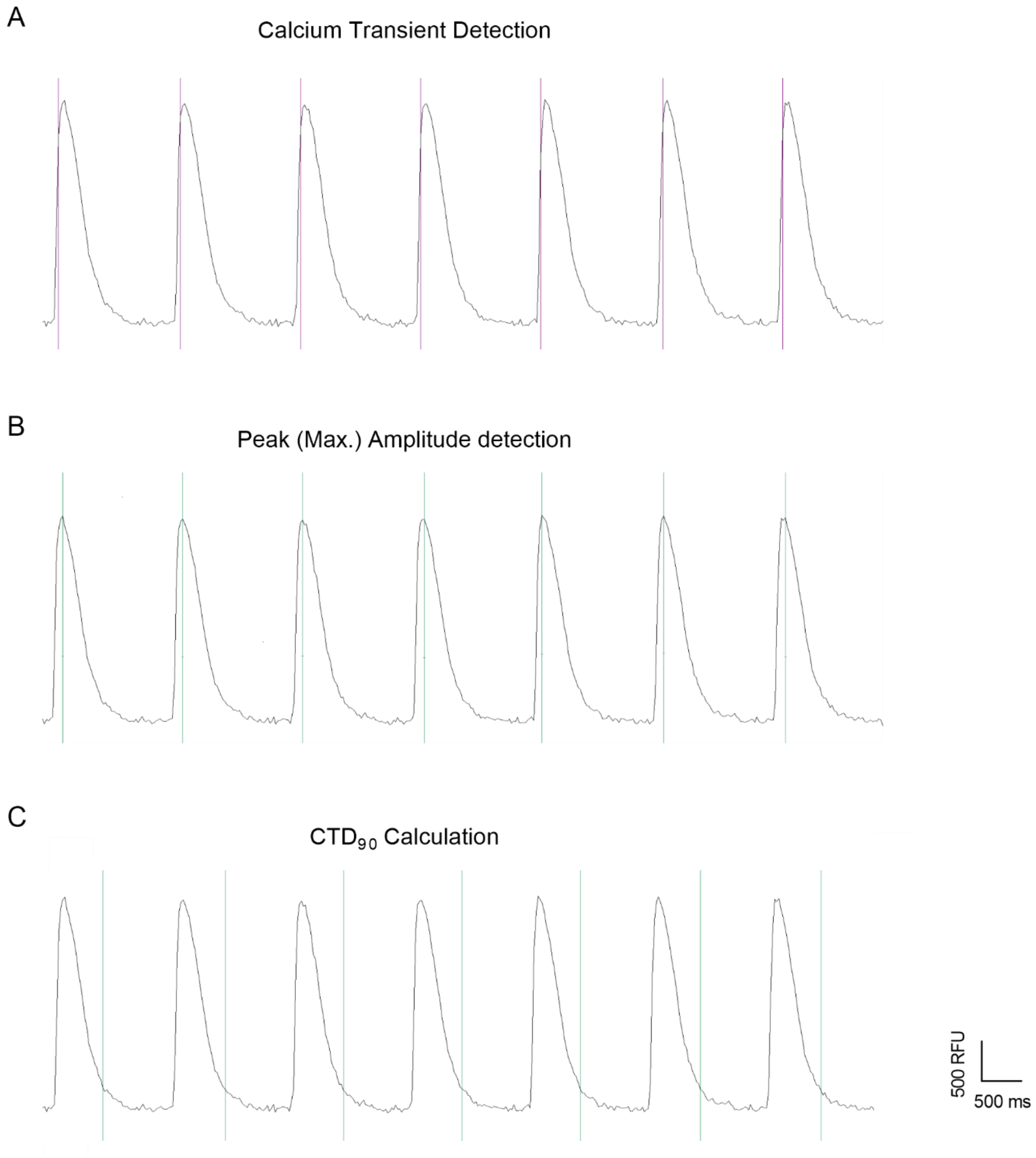

Figure S7. **Calcium transient detection algorithm. Related to Figure 2.** A) Beat/cycle detection. B) Peak amplitude detection. C) CTD_{90} detection/calculation.

Table S1

Compound	Vendor	Product nr.	Lot nr.	Reference free Cmax
Adenosine	Sigma-Aldrich	D9434	SLBH3471V	not available
Ajmaline	Janssen Research Foundation	not available	not available	Redfern et al. (2003)
Alfuzosin	Sigma-Aldrich	A0232	072M4744V	Schulz et al. (2012)
Aprindine	Sigma-Aldrich	A7606	038K4711V	Harmer et al. (2011)
Aspirin	Sigma-Aldrich	A5376	SLBN2916V	Yao et al. (2008)
Atenolol	Sigma-Aldrich	A7655	BCBR3720V	Schulz et al. (2012)
BaCl2	Sigma-Aldrich	342920	MKBH4234V	not available
BAYK8644	WuXi AppTec	EO7563_3_001	not available	not available
Captopril	Sigma-Aldrich	C4042	BCBP9930V	Yao et al. (2008)
Carbachol	Sigma-Aldrich	PHR1511	LRAA3318	not available
Cetirizine	Sigma-Aldrich	C3618	122M4712V	Redfern et al. (2003)
Chlorpheniramine	Sigma-Aldrich	C3025	not available	Ando et al. (2017)
Citalopram	Sigma-Aldrich	PHR1640	LRAA6012	Harmer et al. (2011)
Dextropropoxyphene	Janssen Research Foundation	not available	not available	Schulz et al. (2012)
Digoxin	Sigma-Aldrich	D6003	100M1327V	Schulz et al. (2012)
Dobutamine	Sigma-Aldrich	D0676	055M4018V	Banner et al. (1991)
Dofetilide	TOSLab Ltd.	3979/1	not available	Redfern et al. (2003)
Dofetilide	Sigma-Aldrich	PZ0016	030M4707V	Redfern et al. (2003)
Ebastine	Sigma-Aldrich	E9531	028K4712V	Redfern et al. (2003)
Encainide	Sigma-Aldrich	E9156	018K4611V	Harmer et al. (2011)
Erythromycin	Sigma-Aldrich	E5389	WXBC1653V	Redfern et al. (2003)
Escitalopram	Sequoia Research Products	SRP01460e	not available	Schulz et al. (2012)
Fampridine	ACROS CHIMICA	104570050	A0303988	Schulz et al. (2012)
Flecainide	WuXi AppTec	EO7972_2_002	not available	Ando et al. (2017)
Fluoxetine	Sigma-Aldrich	PHR1394	LRAA9180	Redfern et al. (2003)
Glucose	Sigma-Aldrich	A9251	SLBL0630V	not available
Ibutilide	Sigma-Aldrich	I9910	035M4776V	Redfern et al. (2003)
ICA-105574	WuXi AppTec	EO7972_3_001	not available	not available
Isoprenaline	TCI Chemicals	I0261	YCMCK-JO	not available
Ivabradine	WuXi AppTec	EO7548_6_001	not available	Camm (2006)
JNJ-303	TOCRIS COOKSON	3899	4A/179434	not available
Levcromakalim	Sigma-Aldrich	P154	BGBC4503	not available
Mallotoxin	Sigma-Aldrich	V4629	MKBV4993V	not available
YKP581	SK LIFE SCIENCE	YKP581	BP-01-01-38	not available
Mepyramine	Fluka	PHR1340	LRAA1092	not available
Mesoridazine	Sigma-Aldrich	M4068	081M4705V	Harmer et al. (2011)
Mexiletine	Sigma-Aldrich	M2727	099K1483	Ando et al. (2017)
Mizolastine	Sigma-Aldrich	CDS021588	1582511	Redfern et al. (2003)
Moxifloxacin	Carbosynth Ltd	FM65095	1506016644m	Harmer et al. (2011)
Nicorandil	Sigma-Aldrich	R5648	SLBL9875V	not available
Nifedipine	Sigma-Aldrich	N7634	MKBR1676V	Harmer et al. (2011)
Nimodipine	Prestwick	Prestw-918	not available	Schulz et al. (2012)
Nisoldipine	WuXi AppTec	EO7548_5_001	not available	Schulz et al. (2012)
Olanzapine	Sigma-Aldrich	O1141	035M4781V	Harmer et al. (2011)
Ouabain	Sigma-Aldrich	O3125	021M1512V	not available
Paroxetine	Johnson and Johnson Pharma	not available	not available	Schulz et al. (2012)
Phenytoin	Sigma-Aldrich	PHR1139	P500169	Harmer et al. (2011)
Pinacidil	Sigma-Aldrich	N3539	051M4729V	Thuillez et al. (1991)
Primidone	Sigma-Aldrich	P7295	not available	Schulz et al. (2012)
Procainamide	Sigma-Aldrich	P9391	SLBG4388V	Redfern et al. (2003)

Propafenone	Sigma-Aldrich	P4670	MKBR4240V	Harmer et al. (2011)
Quinidine	Sigma-Aldrich	not available	not available	Redfern et al. (2003)
Raloxifene	Sigma-Aldrich	R1402	MKBS2409V	Czock et al. (2005)
Ranolazine	Sigma-Aldrich	R6152	not available	Ando et al. (2017)
Salbutamol	Sigma-Aldrich	S8260	071M1166V	Schulz et al. (2012)
Sertindole	Sigma-Aldrich	S8072	BGBC4254V	Redfern et al. (2003)
Sertraline	TCI Chemicals	S0507	R8VYD-EC	Harmer et al. (2011)
Sparfloxacin	Sigma-Aldrich	56968	BCBN3519V	Yao et al. (2008)
Tadalafil	Kemprotec	K-1117	not available	Schulz et al. (2012)
Tedisamil	KALI-CHEMIE	KC8857 A5-1/M	not available	Redfern et al. (2003)
Tegaserod	Sigma-Aldrich	SML1504	016M4704V	Appel-Dingemanse et al. (2002)
Thioridazine	Sigma-Aldrich	T9025	BCBQ9396V	Redfern et al. (2003)
Tolterodine	Sigma-Aldrich	PZ0009	100M4706V	Olsson et l. (2001)
Verapamil	WuXi AppTec	EO7972_1_001	not available	Schulz et al. (2012)
Zatebradine	Sigma-Aldrich	Z0127	081M4613V	Roth et al. (1993)
Ziprasidone	USP	1724408	F1J028	Ando et al. (2017)
Zolmitriptan	Sigma-Aldrich	SML0248	022M4724V	Schulz et al. (2012)

Table S1. Compound purchase information and free C_{max} references. Related to Figure 5.

Supplemental Experimental Procedures

Preparation of drug solutions

Compounds were dissolved in dimethyl sulfoxide (DMSO) to obtain a stock solution of 1000-fold the highest test concentration, which was then further diluted to obtain concentrations of 1000-fold the intended concentration. On the day of experiment, these solutions were further diluted with the supplemented Tyrode's solution. Compound addition was done automatically using the Functional Drug Screen System (FDSS/µCell; Hamamatsu, Japan) head stage by adding 100 µL of the 2-fold compound solution to wells with hiPSC-CMs already containing a volume of 100 µL of the experimental solution, finally reaching the intended test concentration in 0.1% DMSO.

Calcium transient detection algorithm

The analysis algorithm consists mainly of multiple parts (beat/cycle, feature and parameter detection) that are executed sequentially. The first part is a beat/cycle detection algorithm using filtering and auto thresholding techniques. The feature detection algorithm then identifies for each detected beat/cycle the beginning (e.g. minimum) and the top (e.g. maximum) of the calcium transient. The third part is a parameter calculation algorithm which uses the detected features to calculate the amplitude and CTD_{90}. Beat rate is calculated based on the time interval between different calcium transient peaks (max.).

Process of defining the weighted points

Defining of the weighted points was based on certain criteria that should reflect the expected hazard labeling for certain drug classes. Here, we explain the most important criteria (requirements) that we applied to design the weighted points system through an iterative approach. The weighted algorithm was first evaluated on the control drugs (Fig. 4C), followed by validation and further fine-tuning using the 66 reference drugs. The first requirement was to have a unique labeling of tested concentrations where EADs were observed (very high hazard). As such, EADs were given 100 points, whereas the cumulation of all other combinations could never reach the 100 points minimum required for very high hazard labeling.

The next requirement was to account for drug responses which would be considered as high hazard. Strong CTD_{90} prolongations (based on dofetilide and E-4031) which could potentially lead to EADs were given 25 points, which was also the minimal number of points required to receive the high hazard label. Mild CTD_{90} prolongations, which also showed clear changes in BR and Amp together with a certain incidence (35-80%) of BS, were also expected to be identified as high hazard. In case all (or most) of the wells showed BS, there would be no primary parameter data available and therefore a high incidence of BS (>80%) was scored as 25 points to reflect the high hazard of this type of response. Also CTD_{90} shortening in combination with changes in BR, Amp and a certain incidence of BS received high hazard labeling. Furthermore, CTD_{90} shortening in combination with strong BR increase and Amp increase were weighted to receive a high hazard label, since this phenotype is observed with strong adrenergic stimulation. On the other hand, CTD_{90} shortening in combination with strong BR decrease and Amp decrease were weighted to receive a low hazard labeling (unless additional BS incidence was observed), since this phenotype most likely reflects calcium antagonism, to which hiPSC-CMs are particularly sensitive. Fibrillation-like observations were also directly associated with high hazard and therefore given 25 points.

Mild CTD_{90} changes in most cases were expected to be labeled as low hazard. A combination of strong changes in BR and Amp, but without any CTD_{90} changes, were relatively rare but could sometimes be observed with e.g. sodium channel blockers. Therefore, strong BR and Amp changes were weighted to reach an accumulated minimum of 10 points (low hazard). Mild decreases in BR and/or Amp were not identified as a hazard. A combination of mild Amp and BR increase was considered an indication of an adrenergic stimulation and therefore labeled as low hazard.

Tolerance interval calculations

Non-parametric tolerance intervals (TIs) were calculated with Wilks' approach (Wilks, 1941) at 95% confidence level covering 90% of population (more details are provided in Supplemental Information). Non-parametric approach truncates number of the lowest and the highest observed values to obtain interval bounds. Wilks' approach utilizes beta distribution to determine number of observations to be truncated to achieve specified confidence and coverage levels. Truncation is performed symmetrically based on Wilks' approach (same number truncated for the lowest and the highest values). The calculations presented in Figure 3 were done on a subset of vehicles and control drugs (mainly plates where reference drugs were tested) based on data from individual experiments. It is important to note that for the calculation of TIs that are supposed to characterize an "usual population", the data set needs to represent the expected effects. Hence, individual experiments were excluded when they showed an unexpected response in hiPSC-CMs that could be attributed to external causes. One-sided

TIs were calculated for the positive controls, whereas for vehicles two-sided tolerance intervals were applied. Note that there is certain minimal sample size (*n* of experiments) needed for non-parametric TIs based on Wilks' approach to achieve given confidence of 95% (when population coverage is 90%). For one-sided interval, at least 29 samples are required, while two-sided interval needs 46 samples at minimum (Krishnamoorthy et al, 2009).

Supplemental References

Ando, H., Yoshinaga, T., Yamamoto, W., Asakura, K., Uda, T., Taniguchi, T., Ojima, A., Shinkyo, R., Kikuchi, K., Osada, T., et al., (2017). A new paradigm for drug-induced torsadogenic risk assessment using human iPS cell-derived cardiomyocytes. J. Pharmacol. Toxicol. Methods. 84, 111-127.

Appel-Dingemanse, S., Horowitz, A., Campestrini, J., Osborne, S., McLeod, J., (2002). The pharmacokinetics of the novel promotile drug, tegaserod, are similar in healthy subjects—male and female, elderly and young. Aliment. Pharmacol. Ther. 15 (7), 937-944.

Banner, W., D. Vernon, D., D. Minton, S., Dean, J., (1991). Non-linear dobutamine pharmacokinetics in a pediatric population. Crit. Care Med. 19 (7), 871-873.

Camm, A.J., (2006). How does pure heart rate lowering impact on cardiac tolerability? Eur. Heart J. Suppl. 8 (suppl_D), D9-D15.

Czock, D., Keller, F., Heringa, M., Rasche, F.M., (2005). Raloxifene pharmacokinetics in males with normal and impaired renal function. Br. J. Clin. Pharmacol. 59 (4), 479-482.

Harmer, A.R., Valentin, J.P., Pollard, C.E., (2011). On the relationship between block of the cardiac Na(+) channel and drug-induced prolongation of the QRS complex. Br. J. Pharmacol. 164 (2), 260-273.

Krishnamoorthy K., Mathew T. (2009). Statistical Tolerance Regions: Theory, Applications, and Computation. John Wiley & Sons, Hoboken, New Jersey.

Olsson, B., Szamosi, J., (2001). Multiple Dose Pharmacokinetics of a New Once Daily Extended Release Tolterodine Formulation Versus Immediate Release Tolterodine. Clin. Pharmacokinet. 40 (3), 227-235.

Redfern, W.S., Carlsson, L., Davis, A.S., Lynch, W.G., MacKenzie, I., Palethorpe, S., Siegl, P.K.S., Strang, I., Sullivan, A.T., Wallis, R., et al., (2003). Relationships between preclinical cardiac electrophysiology, clinical QT interval prolongation and torsade de pointes for a broad range of drugs: evidence for a provisional safety margin in drug development. Cardiovasc. Res. 58 (1), 32-45.

Roth, W., Bauer, E., Heinzel, G., Cornelissen, P.J.G., Van Tol, R.G.L., Jonkman, J.H.G., Zuiderwijk, P.B.M., (1993). Zatebradine: Pharmacokinetics of a novel heart-rate-lowering agent after intravenous infusion and oral administration to healthy subjects. J. Pharm. Sci. 82 (1), 99-106.

Schulz, M., Iwersen-Bergmann, S., Andresen, H., Schmoldt, A., (2012). Therapeutic and toxic blood concentrations of nearly 1,000 drugs and other xenobiotics. Crit. Care 16 (4), R136.

Thuillez, C., Pussard, E., Bellissant, E., Richer, C., Kechrid, R., Giudicelli, J.F., (1991). Arterial vasodilating profile and biological effects of pinacidil in healthy volunteers. Br. J. Clin. Pharmacol. 31 (1), 33-39.

Wilks, S.S., (1941). Determination of Sample Sizes for Setting Tolerance Limits. Ann. Math. Statist. 12 (1), 91-96.

Yao, X., Anderson, D.L., Ross, S.A., Lang, D.G., Desai, B.Z., Cooper, D.C., Wheelan, P., McIntyre, M.S., Bergquist, M.L., MacKenzie, K.I., et al., (2008). Predicting QT prolongation in humans during early drug development using hERG inhibition and an anaesthetized guinea-pig model. Br. J. Pharmacol. 154 (7), 1446-1456.
